# Analysis of 2009–2012 Nutrition Health and Examination Survey (NHANES) Data to Estimate the Median Water Intake Associated with Meeting Hydration Criteria for Individuals Aged 12–80 Years in the US Population

**DOI:** 10.3390/nu11030657

**Published:** 2019-03-18

**Authors:** Jodi Dunmeyer Stookey

**Affiliations:** Children’s Hospital & Research Center Oakland, Children’s Hospital Oakland Research Institute, Oakland, CA 94609, USA; jodidstookey@gmail.com

**Keywords:** total water intake, drinking water, hydration, serum sodium, urine osmolality

## Abstract

In 2005, US water intake recommendations were based on analyses of Nutrition Health and Examination Surveys (NHANES) III data that examined if hydration classification varied by water intake and estimated the median water intake associated with hydration in persons aged 19–30. Given the upcoming 2020–2025 Dietary Guidelines review, this analysis addressed the same two aims with 2009–2012 NHANES data. Methods were updated by defining hydration criteria in terms of multiple measures (serum sodium 135–144 mmol/L and urine osmolality < 500 mmol/kg), expressing water intake as ml/kg, distinguishing plain water intake (PWI) from total water intake (TWI), using weighted age- and sex-specific multivariable models to control for determinants of water intake requirements, and selecting two study samples (the non-acutely ill US population and a sub-group without selected chronic disease risk factors). In the US population and sub-group, the relative risk (RR) of meeting the hydration criteria was significantly greater for individuals with TWI ≥ 45 mL/kg or PWI ≥ 20 mL/kg (for the US population 19–50 years of age: adjusted RR = 1.36, 95% CI: 1.10–1.68 for males; adjusted RR = 1.70, 95% CI: 1.49–1.95 for females. For the sub-group 51–70 years of age: adjusted RR = 2.20, 95% CI: 1.15–4.18 for males; adjusted RR = 2.00, 95% CI: 1.18–3.40 for females). The median (SE) TWI and PWI associated with meeting the hydration criteria for males and females 19–50 years of age were 42 (2) mL/kg and 14 (1) mL/kg and 43 (2) mL/kg and 16 (1) mL/kg, respectively. The significant association between water intake and hydration classification differs from the null association underlying the 2005 water intake recommendations and may lead to different reasoning and inferences for the 2020–2025 Dietary Guidelines.

## 1. Introduction

In 2005, the National Academy of Sciences, Institute of Medicine (IOM) released a report establishing Adequate Intake (AI) reference values for water intake for healthy individuals in the United States (US) [1]. The reference values for water intake were based on observational analyses of data from the 1988–1994 National Health and Nutrition Examination Survey (NHANES) III, because high-quality experimental data were lacking regarding the dose–response effects of water intake on clinically relevant biological outcomes in normal, healthy individuals, under conditions of daily life [1]. The NHANES III data analyses examined whether hydration status varied with water intake and estimated the median water intake associated with adequate hydration. Although new evidence has accumulated, since 2005, data from randomized controlled trials on long-term dose–response effects of water intake on chronic disease risk remain sparse. In preparation for the 2020–2025 Dietary Reference Intakes (DRI) review process, the present study again looks to NHANES data to consider if hydration classification varies with water intake and estimate water intake associated with hydration in healthy individuals in the US.

The NHANES III data analysis operationalized scientific consensus, summarized in the 2005 report [1], about serum osmolality as a primary marker of total body water deficit, water balance regulation being a function of total water intake, and total body water deficit causing acute health effects. The analyses defined hydration in terms of serum osmolality, expressed water intake in terms of L/d total water intake, and selected a study population that was representative of non-acutely ill US men and women, ages 12–18, 19–50, 51–70, and 71–80 years.

Since 2005, researchers have debated the relevance of serum osmolality as a primary hydration biomarker for healthy, free-living people. A point of consensus that has emerged, despite ongoing debate, is that serum osmolality has limited variability in healthy individuals under conditions of normal daily life, because the renin–angiotensin system compensates for inadequate water intake by concentrating urine to retain body water and compensates for excess water intake by excreting dilute urine [2,3,4,5]. Researchers further agree that there is no one gold-standard biomarker of hydration and recommend the use of multiple criteria simultaneously to assess hydration [2,6].

The 2005 IOM report recognized potential for extreme variability in water intake requirements, both between- and within-person, and over time, depending on many factors, including age, sex, body size, health status, protein and salt (solute) intake, physical activity, climate and time of day/circadian rhythm [1]. Although the NHANES III data analyses that informed the IOM report were stratified by age and sex, water intake was not expressed relative to body weight, nor were total water intake estimates adjusted for other determinants of water intake requirements [1]. Water intake was expressed in absolute units (L/d) even though a given L/d intake of water can be expected to have different effects on the hydration status of small versus large individuals. Recent NHANES studies suggest that not accounting for body weight and other risk factors may introduce confounding and/or effect modification into hydration studies [7,8]. Urine flow rate and urine osmolality vary systematically by age, sex, race-ethnicity, and body weight [7]. The relationship between water intake and urine osmolality is significantly modified by weight status [8].

The 2005 IOM report [1] described AI reference values for total water intake, as opposed to plain water (drinking water) intake. Total water intake includes water from all sources: drinking water, as well as moisture from all beverages and food. The report aligned NHANES III data pertaining to total water intake with evidence from experimental water balance studies that did not distinguish sources of water. The experiments measured the effects of induced total body water deficits on ad libitum total water intake [1] (p. 87). Since 2005, data have accumulated indicating that different sources of water intake have different effects on total body water distribution [9,10,11,12,13,14,15,16,17] and energy balance [16,17,18], and consequently, different effects on metabolism and chronic disease risk. The data suggest the need for attention to both total water intake and type of water intake.

Hyperosmotic sources of water draw water out of the body into the gut before absorption, resulting in increases in blood osmolality, cell shrinkage, vasopressin release, urine concentration, and body water retention; hypo-osmotic sources of water, in contrast, cause cell swelling, suppression of vasopressin release, and dilute urine [9,10,11,12,13,14,15,16]. Experiments that report no effect of beverage osmolality on urine volume or osmolality test a large volume of hyperosmotic beverage (a bolus of 1 L consumed in 30 min or 35 mL/kg) and a long delay (24 h) for the water from the beverage to be absorbed and distributed in the body [12,19,20]. The retained body water, while restricted to the extracellular compartment in the short-term, is not associated with improved physical performance [15,21]. The data imply that the osmolality of the water source may modify the relationship between total water intake and hydration and that the osmolality of the water source may modify relationships between total water intake and physical outcomes. Although food and beverages, including milks, juices, and sodas do contribute water to total water intake when consumed, these sources of water are hyperosmotic relative to normal blood osmolality (osmolality > 280 mmol/kg) [22].

Hypo-osmolality downregulates gluconeogenesis, glycogenolysis, and proteolysis, and improves insulin sensitivity [23,24,25,26,27,28,29]. Hypo-osmolality can increase sympathetic nerve activity [30], stimulate the mitochondrial respiratory chain [31], shift the mitochondrial and cytosolic NADH systems to a less reduced state [32], and/or enhance flux through the pentose phosphate shunt [28]. After overnight fluid restriction, a bolus of 500 mL or 10 mL/kg drinking water has been shown to increase energy expenditure in adults and children who are overweight or obese [33,34]. Drinking enough water to suppress vasopressin or dilute urine is associated with greater weight loss for individuals consuming a hypocaloric or low glycemic diet [35,36,37,38], reduced risk of hyperglycemia or diabetes incidence [39,40], and reduced risk of chronic kidney disease [41,42,43,44,45].

Independent of the osmolality or tonicity of the water source, the macronutrient content of the water source determines the effect of the water source on total energy intake. Over 60 randomized experiments report that drinking water instead of a caloric beverage with a meal lowers total energy intake under ad libitum conditions [18]. In randomized weight change trials involving ad libitum conditions, drinking water instead of sugar-sweetened beverages and juice [46,47] or skim milk [48] results in significantly less weight gain. In observational prospective data from the Nurse’s Health Study, drinking water instead of sugar-sweetened beverages is associated with reduced risk of incident type 2 diabetes [49].

Clinicians recognize disorders of body water distribution, hyponatremia, and hypernatremia, as common among inpatients and outpatients and risk factors for increased morbidity and mortality [50]. Clinicians recommend evaluating body water deficit and distribution, together, and tailoring treatment to the underlying cause. Serum sodium and urine osmolality are measures recommended for differential diagnosis of disorders involving the renin–angiotensin system, including hyponatremia and hypernatremia [50,51,52]. Treatment of hypovolemic disorders involves calculating the total free water deficit, based on kilograms body weight and serum sodium, and providing hypotonic free water [50]. The NHANES data have yet to be aligned with clinical practice through observational analyses that involve kilograms body weight, serum sodium, and urine osmolality to classify hydration in terms of both body water volume and distribution, as well as both total water intake and plain water intake.

The overall goal of the present 2009–2012 NHANES data analysis was to determine if hydration classification varied by water intake and to describe the range of water intake associated with meeting hydration criteria for healthy individuals in the US. Taking the new literature into account, the present analysis defined hydration in terms of serum sodium and urine osmolality, using cutoffs that reflect hypotonic cell swelling, expressed water intake per kg body weight, and distinguished plain water intake (PWI) from total water intake (TWI). The analysis focused on non-acutely ill individuals ages 12–80 years, as well as the sub-group of non-pregnant, non-acutely ill individuals who did not have the following risk factors for chronic disease when they presented at the Mobile Examination Clinic (MEC) examination: overweight or obesity, hyperglycemia or elevated hemoglobin A1C, insulin resistance, dyslipidemia, albuminuria or low glomerular filtration rate.

## 2. Materials and Methods

### 2.1. Study Design

This observational study used nationally representative data from the 2009–2010 and 2011–2012 NHANES for retrospective analyses of hydration status at a point-in-time in relation to previous day water intake pattern. The data source and stratifications for this analysis were selected to correspond with the data source and analyses that informed the 2005 IOM report [1].

For the 2009–2010 and 2011–2012 NHANES, participants completed a home interview and a visit to the NHANES Mobile Examination Clinic (MEC). Information about the previous day’s dietary intake, body measurements, health status, and laboratory data were collected at the MEC under controlled, standardized conditions. The MEC visits were scheduled 5 days per week on a rotating schedule that allowed appointments to be scheduled on any day of the week. Participants were randomly assigned to have a MEC visit in the morning, afternoon or evening.

The NHANES datasets that included information about the timing of the MEC visit, participant demographic characteristics, total nutrient intake on the day before the MEC visit, body composition, serum chemistry panel, urine osmolality, urine albumin and creatinine, fasting glucose and insulin, glycated hemoglobin, fasting HDL-cholesterol, fasting triglyceride, physical activity, smoking, diabetes and kidney ever diagnoses, and prescription medication were downloaded in .xpt format from the NHANES website (https://wwwn.cdc.gov/nchs/nhanes/). The files were imported, sorted, and merged by seqn id using Stata software (College Station, TX, USA: StataCorp LP). Data from the 2009–2010 and 2011–2012 NHANES were appended to increase the sample size available for analyses stratified by population sub-group. In 2009–2012, 20,293 individuals participated in NHANES.

### 2.2. Study Participants

The NHANES sample was designed to represent the total civilian non-institutionalized population in the US. Participants were recruited via stratified multi-stage, unequal probability cluster sampling. Each participant was assigned a numerical sample weight that reflects the number of people in the population represented by that specific person. The sample weights were calculated to account for survey non-response, over-sampling, post-stratification, and sampling error. Details about participant recruitment, consent, and weighting are available at the NHANES website [53].

For the present analysis, two samples were drawn from the full NHANES sample. The first study population was drawn to parallel the analyses done for the 2005 IOM report [1], which included all non-acutely ill males and females ages 12–80 years who participated in the NHANES home interview, MEC examination, and Day 1 dietary recall. The second sample was a subgroup of the first, restricted to individuals who were both non-acutely ill and did not have selected chronic disease risk factors at the MEC examination.

In 2009–2012, 12,980 adolescents and adults completed the Day 1 diet recall at the MEC and were eligible for this analysis. Individuals were excluded from the study population if they reported below 0.5 L/d or above 10 L/d total water intake (*n* = 100) or were missing the variables necessary for hydration classification, namely, serum sodium (*n* = 849) or body weight data (*n* = 107). Missing urine osmolality data were not treated as an exclusion criterion to avoid systematically excluding individuals unable to void due to sub-optimal hydration. Complete data were available for 11,924 participants, ages 12 to 80 years, regarding all covariates described below, except for smoking. Questions about smoking were only asked to adults age 20 or older (*n* = 9,800).

Participants included in the first sample (referred to below as the US population) were eligible to be in the sub-group sample. Participants were excluded from the sub-group if they did not fast for 8 hours or longer before the MEC visit (*n* = 6320), were missing height or body mass index (BMI) information (*n* = 4), were underweight (*n* = 96), overweight or obese (*n* = 3652), or met criteria for insulin resistance (*n* = 32), elevated fasting glucose (*n* = 70), elevated glycated hemoglobin (*n* = 19), elevated triglycerides (*n* = 180), low HDL cholesterol (*n* = 193), stage 3–5 chronic kidney disease (CKD) (*n* = 45) or albuminuria (*n* = 122) (see below for details about cutoffs for each measure). Participants were excluded if they self-reported ever being told by a doctor that they had diabetes (*n* = 4) or weak or failing kidneys (*n* = 9). Participants were excluded if they had extreme serum creatinine values (<0.5 or over 1.5 mg/dL) (*n* = 37) or were pregnant (*n* = 6) or lactating (*n* = 5). Due to the small number of individuals ages 71–80 years old remaining in the sub-group, the sub-group was restricted to ages 12–70 years. The sub-group without selected chronic disease risk factors included 1066 participants.

### 2.3. Measures

Details about the MEC staff qualifications and training, the data collection protocol, and laboratory methods used in the 2009–2010 and 2011–2012 NHANES cycles are available at the NHANES websites [54,55].

#### 2.3.1. Demographic Data

Information about each participant’s age in years, sex, race–ethnicity, pregnancy, and six-month period when they visited the MEC were collected by questionnaire during the home interview. Four age groups were defined to match the age groups studied for the 2005 IOM report [1] (Table G-1 on p. 534–536): 12–18 years, 19–50 years, 51–70 years, and 71–80 years. The online NHANES data report race–ethnicity in five categories: Mexican American, Other Hispanic, non-Hispanic White, non-Hispanic Black or Other/Multiracial. This analysis combined Mexican American and Other Hispanic into one group.

#### 2.3.2. Anthropometric Data

All NHANES participants who attended the MEC examination were eligible for body weight and height measurements by a trained health technologist and recorder working as a team in a private MEC exam room [56]. The measurements were completed using a calibrated digital floor scale (Mettler Toledo Panther) that was built into the MEC exam room floor and a calibrated stadiometer. The maximum capacity of the digital weight scale was 440 pounds. The stadiometer had a fixed vertical backboard and an adjustable head piece. Participants were measured wearing the standard MEC examination gown (disposable shirt, pants, and slippers), after removing shoes and any items from their pockets and after removing hair ornaments, jewelry or braids from the top of the head. For weight measurement, the health technologist asked the participant to stand in the center of the scale, with hands at sides, looking straight ahead. The recorder recorded weight measurements in kilograms to one decimal point. For height measurement, participants were instructed to stand on the stadiometer with heels together and toes pointing slightly outward at about a 60 degree angle. The health technologist checked that the back of the participant’s head, shoulder blades, buttocks, and heels made contact with the backboard, aligned the participant’s head so that they looked straight ahead, parallel to the floor and perpendicular to the backboard, lowered the stadiometer head piece to rest firmly on top of the participant’s head, and instructed the participant to take a deep breath. Height measurements were recorded to the nearest tenth of a centimeter and verified by the recorder. The scale and stadiometer were linked electronically to the NHANES Integrated Survey Information System for quick and accurate data entry.

The body mass index (BMI) was calculated from the measured height and weight. Centers for Disease Control & Prevention (CDC) cutoffs were used to define underweight (BMI percentile < 5 for ages 12–19 years and BMI < 18.5 for ages 20 years or older) and overweight or obesity (BMI percentile ≥ 85 for ages 12–19 years and BMI ≥ 25.0 for ages 20 years or older) [57,58].

#### 2.3.3. Dietary Intake Data

A 24-h dietary recall was administered to each NHANES participant, in-person, during the MEC visit in a private dietary interview room. The interview room was equipped with a computer with United States Department of Agriculture (USDA) Automated Multiple Pass software, food models, and 3-dimensional measuring guides, including glasses, bowls, mugs, mounds, circles, thickness sticks, spoons, a ruler, cartons, and 5 different sizes of water bottles. The MEC interviewer was specially trained to explain the purpose of the diet recall, describe what to expect during the diet interview, and ask every participant the same scripted questions, in exactly the same way, with a completely neutral attitude. The interviewer began the diet recall by asking each participant to list foods and beverages that they remembered consuming on the previous day. It then probed for frequently forgotten items, such as beverages and snacks, and details about the brand, preparation, and amount consumed. “Please tell me everything you had to eat and drink all day yesterday from midnight to midnight. Include everything you had at home and away, even snacks, coffee, soft drinks, water, and alcoholic beverages. I’ll ask you for specific details and amounts of the foods in a few minutes.” Interviewers probed for anything added to the food or drinks, such as milk added to cereal or coffee. The interviewers asked the participants about their usual use of salt during food preparation and at the table and the type of salt used. The interviewers used food models and measuring guides to help the respondent estimate the portion size of foods and beverages consumed. To probe regarding water intake, interviewers asked: “Did you drink any tap water yesterday, including filtered tap water and water from a drinking fountain?”, “How much tap water did you drink yesterday?” The interviewers recorded water that was added to foods or drinks, including ice. The interview was conducted in English or Spanish. Proxy interviews were done for individuals who could not report for themselves due to age or disability.

In real-time, as the participant reported each food and drink, the MEC interviewer entered each food and drink into the USDA Automated Multiple Pass software, matching it with a food or drink in the USDA nutrient composition database. In addition to tap water, the software captured ice, bottled waters (various brands, plain, spring, mineral, and electrolyte fortified) and carbonated plain waters (sparkling, seltzer, and club soda). The USDA software aggregates the data entered and outputs estimates of the total daily nutrient intake in addition to category-specific nutrient intake. The present analysis used the estimated daily total intakes of water, protein, sodium, and potassium, as well as the category-specific estimate of plain water intake. Total water intake (TWI) and plain water intake (PWI) were expressed in units of liter per day as well as mL per kilogram body weight per day. To index the potential renal solute load, total daily intakes of protein, sodium, and potassium were combined as: (gm protein × 5.8) + mEq (Na + K). The calculated index of potential renal solute load systematically underestimates the dietary solute load, because it does not include chloride, which was not available in the online NHANES data.

#### 2.3.4. Laboratory Data

Urine and blood samples were collected at the MEC. Details about the protocol for specimen collection and processing and data processing and quality control are described elsewhere [59,60]. Participants were asked to void when they arrived at the MEC. If the participant was unable to void or the volume collected was insufficient for planned clinical and laboratory analyses, up to two more voids were collected from the participant during the MEC visit. For each void, the entire volume of the urinary void was collected. The volume and timing of each urine sample was recorded. The volume of the first urine sample was used in the present analysis. For each participant, urine samples were combined and aliquoted such that all urine analytes were done on the composite sample [61,62].

Urine osmolality was measured on fresh urine by freezing point depression osmometer (Osmette II, Model 5005 Automatic Osmometer, Precision Systems Inc., Natick, MA, USA) at the MEC. Urine and serum were tested at the MEC for human chorionic gonadotropin (hCG) (pregnancy test) by chromatographic immunoassay [63,64].

Aliquoted urine was sent to the University of Minnesota, Minneapolis (MN) for determination of urinary albumin and creatinine. Urinary albumin was determined by solid-phase fluorescent immunoassay. Urinary creatinine was determined using a Roche Hitachi Modular P Chemistry Analyzer [65,66]. The random urine albumin/creatinine ratio was calculated. Albuminuria was defined as urinary albumin–creatinine ratio ≥30 mg/g creatinine [67,68]. Pregnancy was confirmed by positive urine test.

Specially trained MEC phlebotomists administered a questionnaire to determine the length of fast and screen participants for conditions that would exclude them from the blood draw. Blood was collected at the MEC from participants who were not hemophiliacs, had not received chemotherapy within 4 weeks, and did not have rashes, gauze dressings, casts, edema, paralysis, tubes, open sores or wounds, shunt or intravenous lines on both arms.

Blood samples were processed, frozen, stored, and shipped to the University of Minnesota, Minneapolis, MN. Whole blood was assayed for Hemoglobin A1c on a Tosoh A1C G7 system by a laboratory that was participating in the National Glycohemoglobin Standardization Program [69,70].

American Diabetes Association cutoffs were used to define elevated glycated hemoglobin (≥6.5%) [71].

Serum samples were analyzed for sodium and creatinine as part of a routine serum biochemistry profile using a DxC800 system [72,73]. Serum creatinine, age, sex, and race were used to estimate the glomerular filtration rate using the CKD-EPI equation [74] which was found to improve risk stratification beyond other equations [75] and is recommended by the National Kidney Foundation [76]. Stage 3–5 chronic kidney disease was defined as estimated glomerular filtration rate (eGFR) < 60 mL/min/1.73 m^2^ [77]. For participants under age 18, pregnancy was confirmed by serum test.

Fasting plasma insulin and glucose tests were performed on blood collected from participants who had a morning MEC visit and had fasted over 8 hours. Anti-coagulated blood was centrifuged to separate the plasma, which was frozen and sent to the NHANES Diabetes Laboratory in Minneapolis Minnesota. Insulin was determined by enzyme-linked immunosorbent assay (ELISA). Glucose was determined by enzymatic method. Fasting triglycerides were measured by enzymatic method by Roche Modular P chemistry analyzer. Fasting HDL cholesterol was measured photometrically. The Homeostasis Model Assessment of Insulin Resistance (HOMA-IR) index was calculated as: (fasting insulin in Uu/mL) x (fasting glucose in mg/dL). Insulin resistance was defined as HOMA-IR ≥ 2.5, consistent with many previous studies of children and adolescents, including NHANES analyses [78,79]. American Diabetes Association cutoffs were used to define elevated fasting glucose (≥126 mg/dL) [71].

Elevated triglycerides (>130 mg/dL for ages 12–19 years and >150 mg/dL for ages 20 years or older) and low HDL cholesterol (<35 mg/dL for ages 12–19 years, <40 mg/dL for adult men and <50 mg/dL for adults women) were defined using age- and sex-specific cutoffs supported by the American Heart Association, American Association of Clinical Endocrinologists, American College of Endocrinology, and National Heart Lung and Blood Institute (NHLBI) [80,81,82].

#### 2.3.5. Hydration Criteria

Serum sodium and urine osmolality were used to classify study participants with respect to hydration. The hydration criteria for this study were serum sodium within the normal range (135–144 mmol/L) [50] and spot urine osmolality < 500 mmol/kg. The cutoffs were selected to be consistent with conditions below the thirst threshold [51], consistent with zero electrolyte free water clearance (assuming equal urine tonicity and plasma tonicity, given a urinary urea constant of 40%) [2], consistent with proposed cutoffs for spot or 24-h urine osmolality [3,83] and consistent with chronic health benefits [3,38,40]. Participants that met both criteria were identified as meeting hydration criteria. Participants that did not meet both criteria were classified as not meeting hydration criteria. Participants who were unable to void or had a first void urine volume below 50 mL were classified as not meeting hydration criteria. Note that the hydration criteria in this analysis represent benchmarks for grouping individuals and are not treated as criteria for as hypohydration, dehydration or any specific clinical diagnosis. The same criteria were applied to all sex and age groups, despite the fact that the meaning of the cutoffs may vary with sex and age.

#### 2.3.6. Physical Activity

The Global Physical Activity Questionnaire (GPAQ) was administered by trained interviewers at the participant’s home using the Computer Assisted Personal Interviewing (CAPI) system [84,85]. The questionnaire estimates the time spent each day or week walking or riding a bike, doing moderate or vigorous activity at work, and/or doing moderate or vigorous recreational activity. The time spent doing activity was converted to metabolic equivalents (MET) per minute by assigning the walking or biking and moderate activities a MET score of 4.0 and the vigorous activities a MET score of 8.0. Physical activity METs were grouped into the following categorical variable: 0–999 min/week, 1000–4999 min/week, and ≥5000 min/week.

#### 2.3.7. Cigarette Smoking

For adults age 20 years or older, trained interviewers asked each participant if they ever smoked at least 100 cigarettes in their life, and if yes, if they smoke cigarettes now [86,87].

#### 2.3.8. Self-Reported Health Conditions

For adults age 20 years or older, trained interviewers asked each participant: “(Other than during pregnancy (if applicable)), have you ever been told by a doctor or health professional that you have diabetes or sugar diabetes? Have you ever been told by a doctor or other health professional that you had weak or failing kidneys? Do not include kidney stones, bladder infections, or incontinence” [88,89,90,91].

#### 2.3.9. Prescription Medication Use

During the home interview, trained interviewers asked each participant the yes or no question: “In the past month, have you used or taken medication for which a prescription is needed? Do not include prescription vitamins or minerals.” A proxy respondent answered for individuals who could not answer the questions themselves [92,93].

### 2.4. Statistical Analysis

Stata/SE 15.1 statistical software (College Station, TX, USA: StataCorp LP) was used for the analysis. The syvset and svy commands available in Stata were used to account for the NHANES survey design characteristics and population weights. The online NHANES 2009–2010 and 2011–2012 datasets each include Day 1 dietary intake weights (wtdrd1) for use with Day 1 dietary intake data as well as fasting sub-subsample weights (wtsaf2yr) for use with data from participants who were measured in the morning after an overnight fast. Given that the 2009–2010 and 2011–2012 data were combined for this analysis, four-year weights were calculated from the two-year weights (½ x wtdrd1 and ½ x wtsaf2yr) [94]. Four-year Day 1 weights were applied to analyses involving the study population. Four-year fasting weights were applied to analyses involving the 8-hour fasted sub-group.

#### 2.4.1. Descriptive Analysis

The US population and sub-group were described in terms of the variables used to select each sample and classify participants with respect to hydration by sex and age group. Weighted geometric means and 95% confidence intervals were estimated for body weight, serum sodium, urine osmolality, urine volume, fasting serum chemistry measures, glycated hemoglobin, urine albumin: creatinine and eGFR for sex and age groups that correspond with life-stage groups studied for the 2005 IOM report [1].

The US population and sub-group were described in terms of determinants of water intake requirements, which were recognized by the IOM report [1], such as body weight status, level of physical activity, and solute load, and which may provide key background context, confound, and/or modify relationships between water intake and hydration. The weighted proportion of the US population with each determinant of water intake requirements was estimated by sex and age group. Similarly, the weighted proportion of the sub-group with each determinant of water intake requirements was estimated by sex and age group.

The bivariate relationships between serum sodium and urine osmolality and between TWI and PWI were described by scatterplot for the US population and sub-group by sex. The weighted proportion of the US population that met the hydration criteria was estimated by sex and age group. The weighted proportion of the sub-group that met the hydration criteria was also estimated by sex and age group.

Weighted predicted probabilities of meeting the hydration criteria associated with different levels of TWI or PWI were estimated by sex and age group for the US population and sub-group. The independent variable was specified as TWI or PWI to allow for potential threshold, intercorrelated, and/or modifying effects of the TWI and PWI variables. The probabilities were predicted from a series of logistic regression models, all with similar specification. In all of the models, the dependent variable was a dichotomous indicator for meeting the hydration criteria. The independent variable was a dichotomous variable representing whether or not a particular level of TWI or a particular level of PWI was met. Weighted predicted probabilities were estimated for TWI ranging from 20 to 60 mL/kg and PWI ranging from 0 to 50 mL/kg. All logistic regression models controlled for age in years, body weight, solute load, race–ethnicity, level of physical activity, current smoking, prescription medication use in the past month, fasting status, and season of the MEC visit. The margins command available in Stata was used to predict the weighted probabilities, holding each covariate at its mean for each sex and age group. The predicted probability curves were visually inspected to arbitrarily select cutoffs for TWI and PWI that might represent thresholds for effect, distinguishing lower versus higher relative risk of meeting the hydration criteria.

#### 2.4.2. Determine If the Hydration Classification Varied by Water Intake

Poisson models with robust error variances were next used with the arbitrarily selected TWI and PWI cutoffs to test if the relative risk of hydration classification varied by TWI or PWI and TWI and PWI classification in the US population and sub-group by sex and age group, adjusting for covariates. The Poisson models were fit using the svy: glm command with link(log) and eform options available in Stata, as recommended by Lindquist [95]. The Poisson models with TWI or PWI as independent variables were specified as described above for the logistic regression models. Models with TWI and PWI as independent variable treated TWI < 45 mL/kg and PWI < 20 mL/kg as the reference group. Models with TWI and PWI as independent variable tested whether the effect of TWI on the relative risk of meeting the hydration criteria depended on the level of PWI. A total of 3 models were fit for each life-stage group. First, an unadjusted model was fit (Model 1) to test for an association between water intake and the relative risk of meeting the hydration criteria, given the distribution of determinants of water intake requirements as they are under free-living conditions. A multivariable model (Model 2) was next used to adjust the estimated association for the following determinants of individual water intake requirements: age, race–ethnicity, physical activity, dietary solute load, current cigarette smoking, prescription medication use, season and timing of survey participation, and fasting state. Lastly, Model 3, added separate control for body weight to check for confounding specifically attributable to this variable.

Linear regression models were used to estimate the weighted mean difference in TWI associated with meeting the hydration criteria for each sex and age group for the US population and sub-group. Zero-inflated negative binomial (ZINB) regression models were used to estimate the weighted mean difference in PWI associated with meeting the hydration criteria. The linear and ZINB regression models (Models 1–3) were specified as described above for the Poisson models.

#### 2.4.3. Estimate the Median Water Intake Associated with Meeting the Hydration Classification

Finally, to identify the median TWI and PWI associated with meeting the hydration criteria, the weighted 10th, 25th, 50th, 75th, and 90th percentiles of the TWI and PWI distributions were estimated for the US population and sub-group by hydration classification, and sex and age group.

## 3. Results

### 3.1. Sample Characteristics

Table 1 describes the US population, in terms of mean serum sodium, urine osmolality and anthropometric variables. In the US population, body weight ranged from 27.7 to 239.4 kg among males aged 12–80 years and from 29.1 to 230.7 kg among females aged 12–80 years. Appendix A describes the US population in terms of determinants of water intake requirements. Over 25% of the males ages 19-50 years in the US population had a body weight of 95 kg or greater. Over 25% of the females ages 19-50 years in the US population had a body weight of 85 kg or greater. The majority of the population was active less than 5000 met/min/wk. Over 25% of the males ages 19-50 years had an estimated dietary solute load over 1000 mOsm/d. About 60% of participants were measured in warmer months: May–October. Approximately 20% of the males and females ages 19–50 years were current cigarette smokers. Over 70% of the adult males and females in the US population had one or more chronic disease risk factor that made them ineligible for the sub-group.

Table 2 describes the sub-group of the US population ages 12–70 years that was not pregnant or lactating and did not have evidence of overweight or obesity, insulin resistance, diabetes, elevated triglyceride, low HDL cholesterol or chronic kidney disease, based on the results of measurements made in the fasting state at a morning MEC examination. Appendix A describes the sub-group in terms of determinants of water intake requirements.

**Figure 1:**
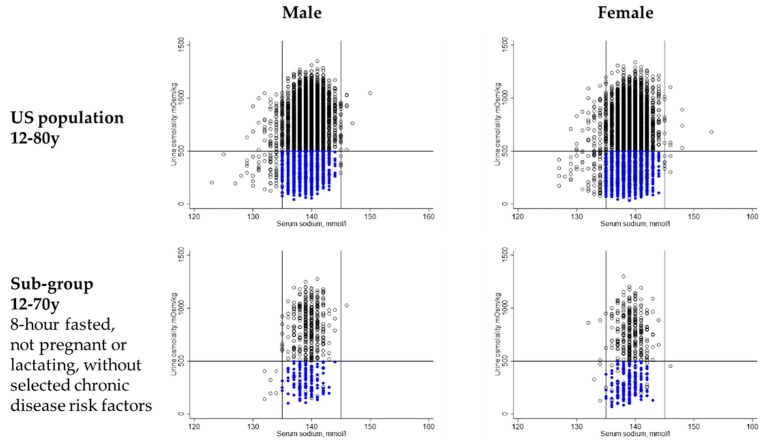
Serum sodium and urine osmolality of males and females in the US population. Each dot represents the serum sodium and urine osmolality of one NHANES 2009–2012 participant. Blue dots represent participants that had both a serum sodium values between 135–144 mmol/L and a urine osmolality below 500 mmol/kg.

Figure 1 describes the distribution of serum sodium and urine osmolality among males and females in the US population and sub-group without selected chronic disease risk factors. In the US population, serum sodium ranged from 123 to 150 mmol/L among males and from 124 to 153 mmol/L among females. Urine osmolality ranged from 44 to 1350 mmol/kg among males and from 34 to 1339 mmol/kg among females.

Under 30% of males and females aged 19–50 years in the US population had a serum sodium within the normal range as well as a urine osmolality below 500 mmol/kg (See Table 3). Approximately half of the males and females aged 51–70 years in the sub-group without chronic disease risk factors met the hydration criteria (See Table 3).

**Figure 2:**
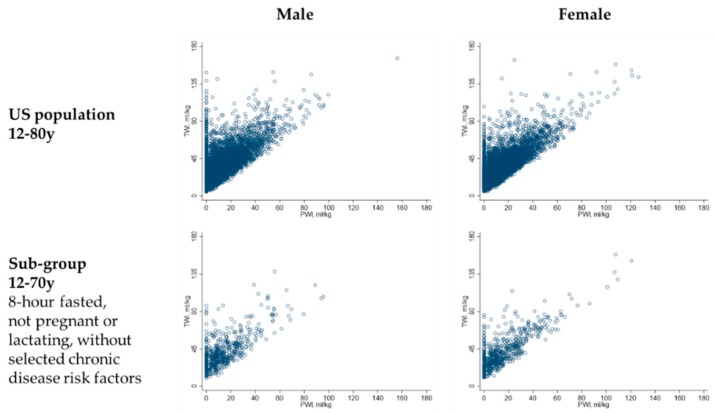
Total water intake (TWI) and plain water intake (PWI) of males and females in the study population. The figure illustrates the correlation between total water intake and plain water intake. Each dot represents the TWI and PWI of one NHANES 2009–2012 participant.

Figure 2 describes the previous day’s TWI and PWI reported by the 2009–2012 NHANES participants. Based on the 10th and 90th weighted percentiles (SE) for the distribution of TWI for each sex in the US population, 80% of males had a TWI between 19.3 (0.4) and 64.0 (1.1) mL/kg and 80% of females had a TWI between 18.0 (0.3) and 61.6 (1.4) mL/kg. An estimated 21.9% (95% CI: 20.1–23.8%) of males and 18.4% (95% CI: 17.0–19.9%) of females reported zero PWI; 99% of males had a PWI below 56.1 (2.5) mL/kg and 99% of females had a PWI below 63.0 (3.4) mL/kg.

**Figure 3:**
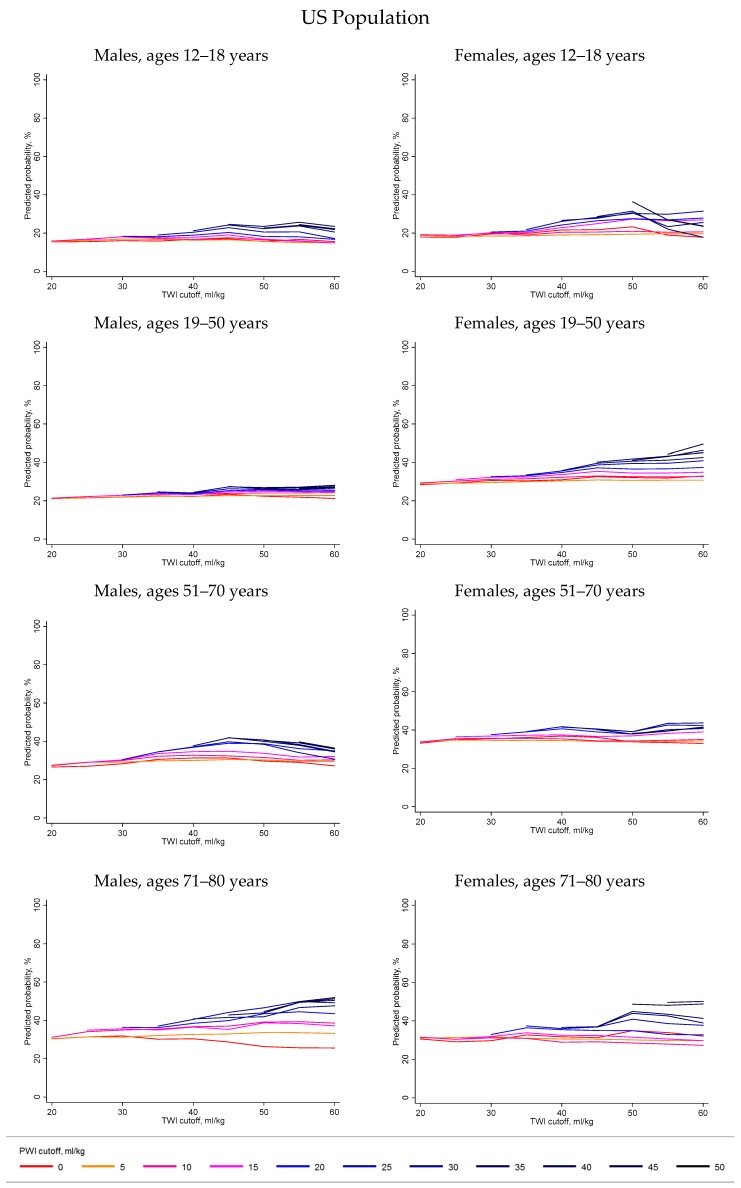

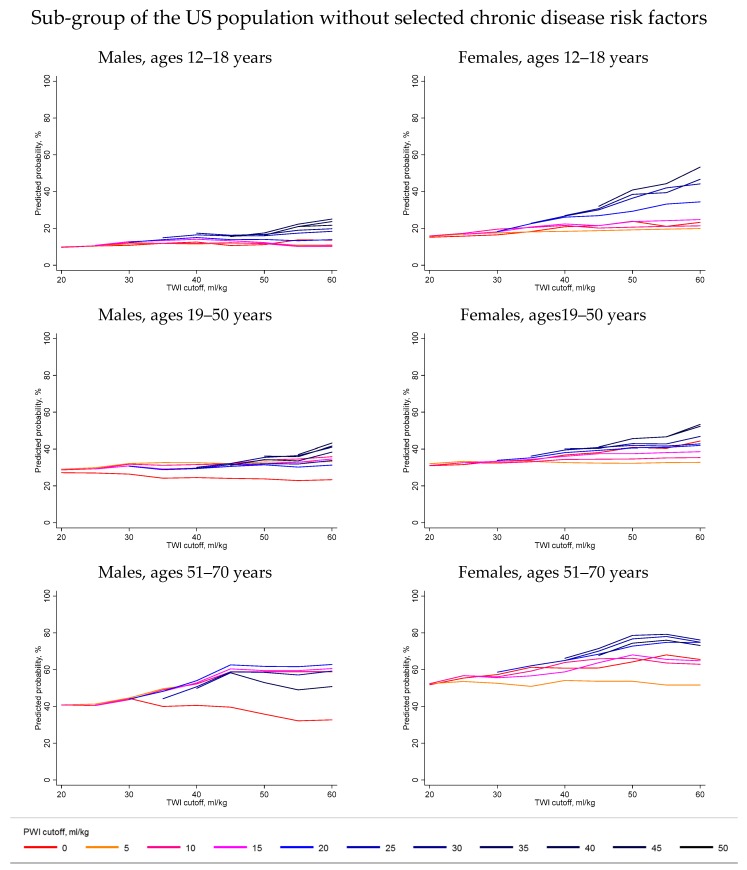
Predicted probability of meeting hydration criteria associated with TWI at or above cutoffs ranging from 20–60 mL/kg or PWI at or above cutoffs ranging from 0–50 mL/kg for the US population and sub-group without selected chronic disease risk factors by sex and age. Participants were classified as hydrated if they had a serum sodium of 135–144 mmol/L, a urine volume ≥ 50 mL, and a urine osmolality < 500 mmol/kg. The probability of meeting the hydration criteria was predicted from weighted, age- and sex-specific logistic regression models that adjusted for age in years, body weight, solute load, race–ethnicity, level of physical activity, cigarette smoking, any prescription medication used in the past month, and season of the MEC visit. The probabilities were predicted holding each covariate at its mean for each sex and age group. See the interactive Tableau dashboard for further detail (Supplementary Figure S1).

Figure 3 summarizes the predicted probability of meeting the hydration criteria associated with TWI cutoffs ranging from 20–60 mL/kg or PWI cutoffs ranging from 0–50 mL/kg for the US population and sub-group without selected chronic disease risk factors. The predicted probability of meeting the hydration criteria and corresponding 95% confidence interval associated with each combination of TWI or PWI is available at https://public.tableau.com/shared/QD2PR2CWP?:display_count=yes. Visual inspection of the predicted probability curves for both the US population as well as the sub-group suggested three things. Firstly, the curves suggested that the probability of meeting the hydration criteria varied by level of water intake. The probability curves were not flat across the levels of TWI. Higher TWI appeared to be associated with a higher probability of meeting the hydration criteria. Secondly, the curves suggested that the probability of meeting the hydration criteria depended on a threshold TWI. Across sex and age groups, around 40–45 mL/kg TWI, the probability of meeting the hydration criteria appeared to plateau. The probability curves did not appear to increase monotonically with TWI. Thirdly, the curves suggested that TWI and PWI had interactive effects on the probability of meeting the hydration criteria. For each age and sex group, the probability curves of different colors did not overlap. For each sex and age group, the red and orange lines, representing the probability of meeting the hydration criteria associated with the lowest levels of PWI, were lower than the dark blue and black lines, representing the probability of meeting the hydration criteria associated with the highest levels of PWI. The probability curves suggested that, given TWI over 40–45 mL/kg, PWI over 20 mL/kg was associated with a higher probability of meeting the hydration criteria than PWI below 20 mL/kg.

### 3.2. Variation in Hydration Classification by Water Intake

Table 4 reports the sex- and age-group-specific weighted relative risk of meeting the hydration criteria at the MEC visit associated with having a previous day TWI at or above 45 mL/kg or a PWI at or above 20 mL/kg for the US population. Without accounting for covariates, i.e., taking the distribution of risk factors, as is, under the free-living conditions of daily life, males and females in the US population, aged 12–18, 19–50, and 51–70 years, with a TWI at or above 45 mL/kg or a PWI at or above 20 mL/kg were over 40% more likely to meet the hydration criteria than males and females with lower TWI and PWI. The sex- and age-specific significant associations were not explained away by control for age in years, race–ethnicity, level of physical activity METs, dietary solute load, cigarette smoking, any prescription medication use, winter season, fasting status, and morning, afternoon or evening timing of the MEC visit. Adjustment for body weight reduced the estimated magnitudes of association by more than 10%, confirming body weight as a potential confounding variable. Controlling for all covariates in Model 3, i.e., assuming a given body weight and set of conditions, TWI at or above 45 mL/kg or a PWI at or above 20 mL/kg was associated with over 25% greater risk of meeting the hydration criteria for the US population ages 12–18, 19–50, and 51–70 years.

Table 4 also reports the sex- and age-group-specific weighted relative risk of meeting the hydration criteria at the MEC visit associated with having a previous day TWI at or above 45 mL/kg or a PWI at or above 20 mL/kg for the sub-group of the US population without selected chronic disease risk factors. The relative risk of meeting the hydration criteria was not significantly associated with TWI at or above 45 mL/kg or a PWI at or above 20 mL/kg in males younger than 51 y. The relative risk of meeting the hydration criteria was significantly associated with TWI at or above 45 mL/kg or a PWI at or above 20 mL/kg in females aged 12–18, 19–50, and 51–70 years, after control for all covariates. In females and males ages 51–70 years, TWI at or above 45 mL/kg or a PWI at or above 20 mL/kg was associated with an over two times greater risk of meeting the hydration criteria.

Table 5 shows the sex- and age-specific weighted relative risk of meeting the hydration criteria associated with levels of TWI and PWI. For males aged 51–70 years and females aged 19–50 years in the US population, TWI at or above 45 mL/kg, *with or without PWI at or above 20 mL/kg*, was associated with significantly increased risk of meeting the hydration criteria compared to TWI less than 45 mL/kg. Among males aged 51–70 years and females aged 19–50 years, without selected chronic disease risk factors, however, only TWI at or above 45 mL/kg, *including PWI at or above 20 mL/kg*, was associated with significantly greater risk of meeting the hydration criteria.

Table 6 reports the predicted mean difference in TWI associated with meeting the hydration criteria versus not meeting the hydration criteria for the US population and sub-group without selected chronic disease risk factors. Total water intake was significantly greater for individuals who met the hydration criteria in all sex and age groups in the US population, with the exception of pregnant women. The significant differences in TWI were not explained away by adjustment for determinants of water intake requirements or body weight. Adjusting for all covariates, the magnitude of difference in TWI ranged from a mean (SE) of 2.8 (1.2) mL/kg in females ages 71–80 years to 8.1 (0.9) mL/kg in females aged 19–50 years. In the sub-group of the US population without selected chronic disease risk factors, TWI was significantly higher for females who met the hydration criteria but did not vary significantly by hydration classification among males.

Table 7 reports the predicted mean difference in PWI associated with meeting the hydration criteria versus not meeting the hydration criteria. In the sub-group without selected chronic disease risk factors, meeting the hydration criteria was associated with a significant mean (SE) difference in PWI of 8.1 (3.0) mL/kg for males aged 51–70 years and 9.6 (4.2) mL/kg for females aged 51–70 years.

### 3.3. Estimate the Median Water Intake Associated with Meeting the Hydration Criteria

Table 8 describes the weighted sex- and age-specific distributions of TWI and PWI in the US population and sub-group by hydration classification. For males aged 19–50 years in the US population who met the hydration criteria, the median (SE) previous day’s TWI and PWI were 3.5 (0.1) L/d and 1.1 (0.1) L/d, which corresponded to 41.9 (1.6) mL/kg and 13.7 (1.1) mL/kg, respectively. For females, aged 19–50 y in the US population who met the hydration criteria, the median (SE) TWI and PWI were 3.0 (0.1) L/d and 1.2 L/d, respectively, corresponding to 42.5 (1.5) mL/kg and 16.2 (1.3) mL/kg.

Among males who did not have selected chronic disease risk factors at age 51–70 years and who met the hydration criteria, the median (SE) previous day’s TWI and PWI were 41.9 (4.2) mL/kg and 14.3 (5.2) mL/kg, respectively. For females without selected chronic disease risk factors at ages 51–70 years, who met the hydration criteria, the median (SE) previous day’s TWI and PWI were 45.9 (4.8) mL/kg and 22.7 (6.0) mL/kg.

**Figure 4:**
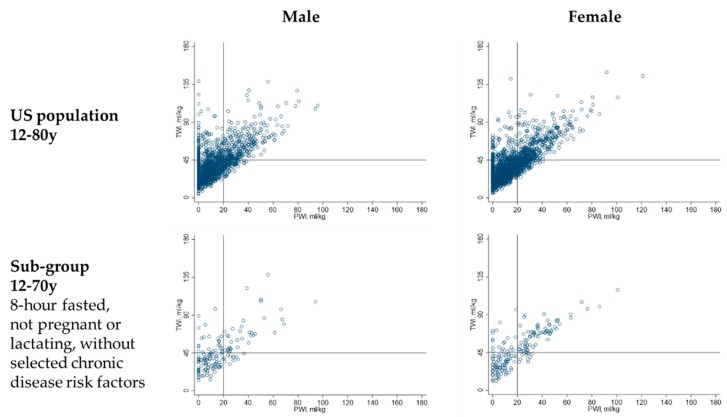
Relationship between TWI and PWI for US males and females who met the hydration criteria in 2009–2012. Each dot represents the total water intake (TWI) and plain water intake (PWI) of one NHANES 2009–2012 participant.

Although some individuals who met the hydration criteria reported zero PWI, higher TWI was significantly linearly associated with higher PWI (see Figure 4). Among males in the US population who met the hydration criteria, each mL/kg increase in PWI was associated with a mean (SE) increase in TWI of 0.9 (0.03) mL/kg (*p* < 0.001). Among females in the US population, each mL/kg increase in PWI was associated with a mean (SE) increase in TWI of 1.0 (0.02) mL/kg (*p* < 0.001).

## 4. Discussion

This paper reports analyses of nationally representative 2009–2012 NHANES data to inform the 2020–2025 Dietary Guidelines. Like the NHANES III data analyses which informed the 2005 IOM water intake recommendations, the present analyses were descriptive in nature and designed to address the same two questions: Did hydration classification vary by water intake? What was the median water intake associated with meeting hydration criteria for healthy individuals in the US?

The NHANES data offer critical information about the level and type of water intake that is available, accessible, affordable, socially desirable, and ultimately feasible for individuals to achieve under free-living conditions in the US, as well as information about the realized effects of water intake on hydration biomarkers, given the background or contextual conditions of daily life in the US. Prevalent factors, such as high salt intake, might modify the effects of water intake on hydration. Observational data from the NHANES make it possible to consider the generalizability of causal, dose–response effects of water, demonstrated in controlled experiments, to the conditions of daily life in the US.

### 4.1. Did Hydration Classification Vary by Water Intake?

Unlike the NHANES III data analyses, which reported no significant variation in hydration by level of TWI [1], the present analyses of 2009–2012 NHANES data showed significant variation in hydration classification by water intake, independent of various determinants of water intake requirements. In the present analyses, the relative risk of meeting the hydration criteria was over 50% greater for males and females ages 19–50 years and 51–70 years with TWI ≥ 45 and PWI ≥ 20 versus TWI < 45 and PWI < 20. The difference in findings reflects the use of different hydration biomarkers. The lack of variation in serum osmolality by TWI in the NHANES III analysis is consistent with tight homeostatic control of serum osmolality.

Beyond indicating that the level of TWI had a significant effect on the likelihood of meeting hydration criteria, the present analyses further suggested that the effect was not monotonic and was dependent on the level of PWI. For each sex and age group, the predicted probability for meeting the hydration criteria appeared to plateau above approximately 45 mL/kg TWI. The probability curves for each sex and age group showed no increase in the predicted probability of meeting the hydration criteria across the range of TWI if PWI was under approximately 10 mL/kg. Further research is needed to check for sex- and age-specific thresholds and interactive effects of TWI and PWI.

In the sub-group without selected chronic disease risk factors, the relationship between TWI and risk of meeting the hydration criteria depended on the level of PWI. Among males and females aged 51–70 years in the sub-group without selected chronic disease risk factors, only a TWI of 45 mL/kg or more, *including PWI of 20 mL/kg or more*, was significantly associated with increased odds of meeting the hydration criteria. The relative odds of meeting the hydration criteria associated with a TWI of 45 mL/kg or more, including less than 20 mL/kg PWI, did not differ significantly from the reference category. The results suggest that individuals who met the hydration criteria via intake of non-PWI sources had one or more chronic disease risk factors, which excluded them from the sub-group. Randomized trials are needed to determine if PWI and non-PWI sources of water are, respectively, effective for meeting water intake requirements at the same time as limiting obesity and chronic disease risk. If non-PWI sources of water intake prove ineffective for meeting both short-term hydration and long-term health requirements, separate recommendations for TWI and PWI might be needed.

### 4.2. Median Water Intake Associated with Meeting the Hydration Criteria

The 2005 IOM report [1] used the median TWI of males and females aged 19–30 years in the NHANES III dataset to set an AI of 3.7 L/day and 2.7 L/day TWI for adult males and females, respectively. The IOM report set the same AI of 3.7 L/day and 2.7 L/day TWI for males and females aged 30–50 years citing “no reason to assume that the level recommended for adults 19–30 years would be in excess [1] (p. 145)” for individuals aged 30–50 years.

In the present analysis, the estimated median TWI of males and females in the US population, aged 19–50 years, who met the hydration criteria, were within 300 mL of the AI set by the 2005 IOM report. The estimated median (SE) TWI for males aged 19–50 years was 3.5 (0.1) L/day. The estimated median (SE) TWI for females aged 19–50 years was 3.0 (0.1) L/day. Expressed relative to body weight, the median (SE) TWIs associated with meeting the hydration criteria were 42 (2) mL/kg and 43 (2) mL/kg for males and females aged 19–50 years, respectively.

For males, the median (SE) TWI associated with meeting the hydration criteria ranged from 40 (4) to 44 (6) mL/kg for age groups 12–18 years, 19–50 years, and 51–70 years in the US population, overall, as well as in the sub-group without selected chronic disease risk factors. The median (SE) PWIs associated with meeting the hydration criteria were 14 (1) mL/kg for males aged 19–50 years in the US population, 14 (2) mL/kg for males 19–50 years in the sub-group, and 14 (5) for males 51–70 years in the sub-group without selected chronic disease risk factors. Based on the standard errors for the estimates, some of the median TWIs and PWIs might be off by as much as 0.5 L, if the mean body weight for males (86.4 kg) aged 19–50 years is multiplied by the standard error of the estimate. Controlled clinical studies would be needed to more precisely determine the TWI and PWI associated with meeting the hydration criteria.

For females, the median (SE) TWI and PWI associated with meeting the hydration criteria appeared slightly higher in the sub-group without selected chronic disease risk factors. For all US females, ages 19-50 and 51-70 years, TWI ranged from 40 (2)−43 (2) mL/kg and PWI ranged from 14 (2)–16 (1) mL/kg. In the sub-group without selected chronic disease risk factors, in contrast, TWI ranged from 46 (5)-48 (6) mL/kg and PWI ranged from 19 (4)–23 (6) mL/kg. The data raise questions about whether or not individuals with chronic disease risk factors differ from those without such risk factors with respect to the median TWI and PWI associated with meeting the hydration criteria. The present study was not designed to test for such a difference. The data, furthermore, raise questions about sustaining a TWI close to 45 mL/kg, including 20 mL/kg PWI, from adolescence into adulthood to increase the relative odds of meeting the hydration criteria, and at the same time, reduce the risk of incident obesity, insulin resistance, diabetes, dyslipidemia, and chronic kidney disease through ages 51–70 years. Multiplying 45 mL/kg TWI and 20 mL/kg PWI by the mean body weight of females (73.3 kg) ages 19–50 years in the US population yields 3.3 L/day and 1.5 L/day PWI for females.

### 4.3. Analysis Strengths

Several aspects of the NHANES data collection protocol support inference about the effects of water intake on hydration status. Although the NHANES is a cross-sectional survey, the timing of data collection establishes the temporality of water intake preceding hydration biomarker assessment. The NHANES collected information about water intake on the day before the MEC examination and blood and urine biomarkers of hydration at the MEC examination. Given that water intake is a function of thirst, which is stimulated by total body water deficit [51], individuals with sub-optimal hydration may appear to have higher water intake in cross-sectional surveys. Prospective experiments are required to unequivocally tease apart endogenous effects of water intake on hydration.

The timing of the MEC data collection in the morning or afternoon/evening was randomized, which evenly distributed variability in water intake and hydration due to circadian rhythm and/or NHANES instructions to restrict food and fluid intake prior to the MEC visit. About 40% of the 2009–2012 NHANES participants were measured after an 8-hour fast. Although the NHANES protocol did not randomize other variables known to determine water intake requirements, it did collect information that enabled multivariable statistical control for between-person differences in water requirements. Randomized controlled experiments would nevertheless be required to rule out confounding by unobserved factors.

### 4.4. Analysis Limitations

The present analysis required several decisions and design limitations, which may impact the interpretation of the results. Decisions were required regarding what NHANES datasets, what population/subgroup(s) to select for analysis, how to define the hydration criteria, how to specify water intake, and how to specify the multivariable models to estimate predicted probabilities and odds ratios.

#### 4.4.1. Choice of Datasets

The present analysis was restricted to data from the 2009–2012 NHANES, which are a decade old and may not represent current conditions. Urine osmolality data were not collected in more recent NHANES cycles. Restriction to only 4 years of data (2009–2012) limited the sample size available for life-stage-specific sub-group analyses. Estimates for pregnant women and the sub-group may be unstable, due to limited sample size.

#### 4.4.2. Choice of Study Population and Sub-Group

To mirror the NHANES III data analyses which informed the 2005 water intake recommendations [1], the present 2009–2012 NHANES data analyses summarized the water intake of all non-acutely ill individuals aged 12–80 years, by sex and age group, without stratifying by myriad determinants of water intake requirements. Results were not presented for each physical activity- and solute-load-specific group, for example. Inferences drawn from the present results about the level(s) of water intake associated with meeting hydration criteria for the US population, therefore assume the underlying distribution of determinants of water intake described in Table 1. As over 80% of the adult men and women in the US population had one or more chronic disease risk factor (see Appendix A), the results presented for the non-acutely ill US population may not reflect water intake requirements in or for optimal health.

The 2005 water intake recommendations were explicitly designed to reduce deleterious *acute* effects of total body water deficit [1]. To enable the 2020–2025 Dietary Guidelines to consider levels of water intake that might also reduce chronic disease risk associated with sub-optimal water intake, the present analysis separately reported results for a sub-group of normal weight individuals who did not have insulin resistance, diabetes, dyslipidemia or chronic kidney disease per laboratory tests at the MEC. Results for the sub-group highlight levels of water intake that were achieved under free-living conditions and associated with the absence of both acute illness and chronic disease conditions. Randomized trials would be needed to test and confirm whether the levels of water intake observed for the sub-group effectively reduce obesity and chronic disease risk.

The 2005 IOM report selected individuals aged 19–30 years who were not acutely ill as a reference group to set the AI [1]. This selection reflects the IOM reasoning that the observed water intake of non-acutely ill, free-living individuals with free access to water is an index of the actual water intake requirements of the individuals, because water intake under those conditions is a function of thirst, and thirst is least impaired in younger adults [1]. An alternative reference group for setting the AI might be individuals aged 51–70 years who are not acutely ill and do not have chronic disease. An alternative reasoning might be that the older age group that has remained free of chronic disease gives a window on what low risk looks like for people in the age group most at risk for chronic disease.

#### 4.4.3. Choice of Hydration Criteria

The choice of hydration biomarkers to use in studies involving conditions of daily life remains controversial [2,3,4,5,6]. Acknowledging the remaining controversy as well as the fact that the biomarkers used in the present study have limitations, the present analysis does not claim to be the best or only way to analyze NHANES data or preclude analyses based on other hydration criteria. The intent of the present analysis is to make available estimates of the prevalence of hydration and level(s) of water intake associated with hydration, if we assume serum sodium and urine osmolality as hydration criteria. The results can be compared with estimates based on serum osmolality and/or other hydration criteria.

The hydration biomarkers in the present analysis are limited in that they are highly sensitive to acute hydration and may misclassify individuals with respect to chronic hydration. Urine osmolality reflects conditions in the 2–4 h before the urine collection [21,96]. One bolus of 500 mL drinking water can acutely decrease urine osmolality from above 800 mmol/kg to below 500 mmol/kg in healthy, fasting young men sitting at rest [97], as well as overweight or obese adolescents sitting at rest [38]. The sensitivity of urine osmolality to water intake in the 2–4 hours before the urine collection limits interpretation of relationships between urine osmolality and previous day water intake (water intake > 12 h prior to collection). For NHANES participants who attended an afternoon or evening MEC visit, interpretation of the relationship between afternoon or evening urine osmolality and the previous day’s water intake depends on the extent to which the previous day’s water intake represents usual water intake. NHANES participants who consumed fluid in the hours preceding their urine collection could conceivably have had unusually dilute urine. Conversely, NHANES participants who did not consume food or fluid because of NHANES protocol instructions to restrict food and beverage intake prior to fasting blood tests could conceivably have had unusually elevated urine osmolality.

Individuals with elevated urine osmolality, who did not meet the hydration criteria in the present study, might nevertheless meet other criteria for chronic hydration, such as saliva osmolality < 100 mmol/kg. In the Adapt Study [98], for example, two out of five participants with serum sodium below 145 mmol/L and urine osmolality over 800 mmol/kg had a saliva osmolality under 100 mmol/kg and appeared chronically hydrated, based on their subsequent response to a sustained 4-week water intake intervention [98]. Unlike participants who initially had a saliva osmolality of 100 mmol/kg or higher, who responded to the higher water intake with gradual isotonic retention of potassium and/or sodium and a 1.8% increase in body weight over 4 weeks, participants with an initial saliva osmolality below 100 mmol/kg decreased the half-life of water in the body in proportion to the increase in water intake, over 4 weeks, and did not increase body weight. In the present analysis, if people who were hydrated were misclassified as not meeting hydration criteria, then the estimated effects of water intake on the relative risk of meeting the hydration criteria may be biased towards the null.

The hydration biomarkers in the present study may not be equally appropriate for all life-stage groups. The hydration criteria in the present study may be more likely to misclassify older adults than younger adults. As urine concentration capacity decreases with age [1], older adults may be more likely to have lower urine osmolality, making them more likely to meet the present hydration criteria. Also, urine osmolality has reportedly limited diagnostic accuracy in older adults in part because they experience a type of fluid disorder (isotonic hypovolemia) that is not detected by urine osmolality [99,100]. Further analyses with other hydration biomarkers are warranted regarding the water intake requirements of older adults.

Aside from what biomarkers to use to define hydration criteria, decisions were required regarding what cutoffs to apply to each biomarker. The present analysis selected a cutoff of 500 mmol/kg for urine osmolality, which is lower than the 800 mmol/kg cutoff frequently applied for this biomarker in studies involving adults and children e.g., [101,102,103,104,105,106,107]. The lower cutoff was selected to focus attention on adequate water to swell cells, suppress vasopressin, and excrete dilute urine. Rather than distinguishing between dehydrated and not dehydrated (which is how urine osmolality ≥ versus < 800 mmol/kg is interpreted [101,102,103,104,105,106,107]) to describe adverse effects of dehydration, the present criteria aimed to distinguish between euhydrated and non-euhydrated to explore for doses of water intake that might be associated with chronic health benefits. Other studies have taken this approach [38]. The term “meet hydration criteria” was deliberately used throughout the paper instead of a term like “euhydration”, because the field has yet to agree on the biomarker(s) and cutoff(s) that define euhydration. The choice of hydration criteria for the present analysis is consistent with a recent proposal to define euhydration in terms of lack of effort or compensation by the body to retain body water [108].

Using serum sodium 135–144 mmol/L and urine osmolality < 500 mmol/kg as hydration criteria, fewer than 30% of the non-acutely ill US population in 2009–2012 met the hydration criteria. The relatively low prevalence is not surprising because having a urine osmolality below 500 mmol/kg, i.e., having adequate water to excrete excess free water, is not regulated like having adequate water to avoid body water deficit. Although thirst guides water intake to restore body water deficit, it does not guide water intake to sustain suppressed vasopressin and suppressed urine concentration throughout the day. Thirst and water intake behavior is triggered by osmoreceptor cell shrinkage and is suppressed by cell swelling [1,51]. Water intake to maintain a urine osmolality below 500 mmol/kg might only occur in the absence of thirst. If thirst is an unlikely driver for water intake to meet the present hydration criteria, then the low prevalence of meeting the hydration criteria would need to be attributable to some other driver(s), such as social policies and norms about when, where, what, and how much to drink and/or barriers to extra water intake, such as lack of time to use the toilet or lack of public restrooms. Future research could check for effects of policies and norms on the likelihood of meeting hydration criteria.

Use of the present hydration criteria assumes adequate solute intake. Researchers caution that large quantities of daily water intake can put healthy people at risk of developing hyponatremia if solute intake is chronically inadequate [109]. The average sodium intakes of US men and women (4107 and 3007 mg per day [110]) exceed the recommended intake of less than 2300 mg per day [111].

#### 4.4.4. Choice of Water Intake Specification

Beyond recognized sources of recall error and bias in self-reported dietary intake data, the choice of water intake specification is a potential source of inconsistent findings across studies. Water intake specified as L/d leads to different results than water intake specified as mL/kg. In the present dataset, for example, for men in the US population ages 19–50 years, TWI expressed in absolute units (L/day) did not differ significantly by hydration classification, while TWI expressed relative to body weight did differ significantly by hydration classification. The estimated unadjusted mean difference in absolute TWI between individuals who met the hydration classification and those who did not was 0.2 (0.1) L/day with a *p*-value of 0.08. In contrast, the estimated unadjusted mean difference in relative TWI was 5.0 (1.4) mL/kg with a *p*-value of 0.001.

Water intake was not expressed relative to solute load in the present analysis, though the dietary solute load from protein and sodium and potassium was estimated and controlled in the multivariable models. The observed effects of water intake and hydration classification in this study may depend on the background dietary intake. As Cheuvront et al. [2] note, achieving an osmolality below 500 mmol/kg can require over 3L/d pure water intake as well as a diet low in protein and salt.

In the present analyses, the significant correlation between TWI and PWI signals potential for relationships between TWI and hydration classification to be confounded by PWI and vice-versa. The estimated differences in TWI associated with meeting the hydration criteria, which are reported in Table 6 of this paper, should be interpreted with the potential confounding by PWI in mind. The regression models that generated Table 6 did not control for the composition of the water sources contributing to TWI. Similarly, the models that generated results in Table 7 did not control for TWI.

The present analysis arbitrarily chose TWI and PWI cutoffs to describe predicted probabilities and compare relative risk of meeting the hydration criteria across groups of participants. To check for a significant difference in the relative risk of meeting the hydration criteria associated with higher versus lower TWI and/or PWI, individuals in all life-stage groups were grouped based on the same 45 mL/kg TWI and 20 mL/kg PWI cutoffs, corresponding to the approximate median intake levels for the sub-group of females ages 51–70 years. To identify TWI and PWI cutoffs that are optimally sensitive and specific for meeting hydration criteria for individuals in each life-stage group, further ROC and signal detection work would be warranted. Describing the sensitivity and specificity of TWI and PWI cutoffs was not an aim of the NHANES III analysis linked to the 2005 IOM report [1], so was beyond the scope of the present 2009–2012 NHANES analysis.

#### 4.4.5. Choice of Multivariable Model Specification

The analysis predicted the probability of meeting the hydration criteria associated with specified levels of TWI or specified levels of PWI for each sex and age group, holding each covariate at the study population or sub-group mean value. The analyses described the probability of meeting the hydration criteria, given the underlying distribution of water intake determinants. The present analysis did not, however, explore the predicted probabilities, assuming alternative underlying water intake determinants. Future studies might predict the probabilities and relative risk of meeting the hydration criteria holding covariates at specified combinations of body size, race–ethnicity, physical activity, dietary solute intake, smoking, prescription medication use, diuretics, and health status.

## 5. Conclusions

This analysis of 2009–2012 NHANES data attempted to follow in the footsteps of the NHANES III data analysis that informed the 2005 IOM report [1]. The present analysis operationalized current scientific consensus regarding use of multiple hydration biomarkers, need to control for between-person differences in individual water intake requirements, assessment of the amount as well as type of water, and sub-optimal water intake causing acute as well as chronic health effects. The analyses updated the NHANES III data analysis by defining hydration in terms of serum sodium and urine osmolality, expressing water intake in terms of mL/kg body weight, distinguishing between PWI and TWI, using multivariable models to control for determinants of water intake requirements, and selecting a study population that was representative of US men and women that was non-acutely ill as well as free of selected chronic disease risk factors. The analyses make 2009–2012 NHANES data available for consideration during the 2020–2025 Dietary Guidelines review process.

Unlike the NHANES III analysis, which suggested that hydration status in the US population does not vary by level of TWI [1], the present analysis suggests that hydration classification does in fact vary by level of TWI and PWI. The conflicting result implies need to reconsider the assumption made for the 2005 report [1] that individuals in the US population meet water requirements by consuming any level of water intake, following thirst.

The present analyses described the distribution of TWI and PWI associated with meeting specific hydration criteria. The results direct attention to TWI at or above 45 mL/kg or PWI at or above 20 mL/kg for simultaneously meeting the hydration criteria and limiting chronic disease risk. The results call for controlled clinical trials to search for precise, optimal cutoffs for each population sub-group. The results suggest opportunity to express water intake recommendations in terms of mL/kg, address confusion about the need for 8 x 8 glasses of water [112] and give the public options for meeting water requirements via TWI or PWI.

## Figures and Tables

**Table 1:** Characteristics of the US population aged 12–80 years in 2009–2012.

	Age 12–18 Years	Age 19–50 Years	Age 51–70 Years	Age 71–80 Years
	Weighted Mean (SE)	Weighted Mean (SE)	Weighted Mean (SE)	Weighted Mean (SE)
Male	(*n* = 978)	(*n* = 2703)	(*n* = 1551)	(*n* = 713)
Body weight, kg	66.8 (0.9)	86.4 (0.5)	89.0 (0.8)	81.9 (0.5)
Height, cm	170.0 (0.5)	176.5 (0.3)	175.7 (0.4)	172.1 (0.3)
BMI	23.1 (0.3)	27.7 (0.2)	28.8 (0.3)	27.6 (0.2)
Serum Na, mmol/L	139.4 (0.1)	139.2 (0.1)	139.1 (0.1)	139.5 (0.1)
Urine Volume, mL	99.3 (4.8)	113.7 (2.9)	100.1 (3.2)	77.4 (2.9)
Urine Osmolality, mmol/kg	721 (24)	618 (10)	566 (9)	550 (8)
Urine Albumin:Creatinine	7.7 (0.5)	5.4 (0.2)	7.9 (0.4)	15.6 (0.9)
eGFR, mL/min/1.73 m^2^	135.8 (1.1)	102.3 (0.5)	81.5 (0.7)	62.5 (0.9)
Serum Creatinine, mg/dL	0.8 (0.01)	0.9 (0.01)	1.0 (0.01)	1.1 (0.01)
HgA1c, %	5.2 (0.01)	5.4 (0.01)	5.8 (0.03)	5.9 (0.03)
Female	(*n* = 882)	(*n* = 2849)	(*n* = 1526)	(*n* = 722)
Body weight, kg	59.4 (0.8)	73.3 (0.5)	74.9 (0.8)	68.7 (0.8)
Height, cm	161.0 (0.3)	163.0 (0.3)	161.5 (0.3)	157.7 (0.3)
BMI	22.9 (0.3)	27.6 (0.2)	28.7 (0.3)	27.7 (0.3)
Serum Na, mmol/L	139.2 (0.1)	138.6 (0.1)	139.5 (0.1)	139.2 (0.1)
Urine Volume, mL	74.3 (3.6)	78.6 (2.1)	73.7 (3.3)	54.4 (2.3)
Urine Osmolality, mmol/kg	620.5 (15.1)	521.9 (12.2)	455.2 (11.5)	455.9 (8.9)
Urine Albumin:Creatinine	12.0 (0.9)	7.7 (0.1)	9.0 (0.4)	16.3 (1.0)
eGFR, mL/min/1.73 m^2^	128.3 (2.3)	106.3 (0.5)	82.9 (0.7)	60.9 (0.9)
Serum Creatinine, mg/dL	0.7 (0.01)	0.7 (0.004)	0.8 (0.01)	0.9 (0.01)
HgA1c, %	5.3 (0.02)	5.3 (0.01)	5.8 (0.02)	5.9 (0.03)

Data representative of the US population were obtained from the National Health and Nutrition Examination Surveys (NHANES). The study population included all participants in the 2009–2012 NHANES with data for water intake, serum sodium, and body weight. Estimates in this table are geometric means and standard errors (SE) with survey weights applied. BMI: Body mass index; eGFR: estimated glomerular function; HgA1c: Glycated hemoglobin.

**Table 2:** Characteristics of the US population sub-group aged 12–70 years in 2009–2012 without selected chronic disease risk factors.

	Age 12–18 Years	Age 19–50 Years	Age 51–70 Years
	Weighted Mean (SE)	Weighted Mean (SE)	Weighted Mean (SE)
Male	(*n* = 201)	(*n* = 233)	(*n* = 96)
Body Weight, kg	58.7 (0.9)	69.4 (0.7)	70.7 (1.0)
Height, cm	170.6 (0.8)	176.4 (0.5)	176.8 (0.9)
BMI	20.2 (0.2)	22.3 (0.2)	22.6 (0.2)
Serum Na, mmol/L	139.7 (0.1)	139.2 (0.2)	138.9 (0.3)
Urine Volume, mL	77.2 (7.6)	110.3 (9.4)	86.5 (15.3)
Urine Osmolality, mmol/kg	731 (28)	561 (32)	508 (27)
Urine Albumin:Creatinine	6.7 (0.6)	5.1 (0.2)	5.5 (0.3)
eGFR, mL/min/1.73 m^2^	137.3 (1.1)	108.3 (1.3)	86.7 (1.4)
Serum Creatinine, mg/dL	0.8 (0.01)	0.9 (0.01)	0.9 (0.02)
HgA1c, %	5.2 (0.03)	5.3 (0.03)	5.5 (0.04)
Fasting HOMA-IR	0.8 (0.04)	0.6 (0.03)	0.6 (0.03)
Fasting Glucose, mg/dL	93.3 (0.5)	94.0 (0.7)	97.1 (1.6)
Fasting Triglyceride, mg/dL	57.3 (2.0)	78.0 (2.4)	82.8 (3.9)
Fasting HDL, mg/dL	53.7 (0.7)	55.0 (0.9)	58.8 (2.3)
Female	(*n* = 132)	(*n* = 305)	(*n* = 98)
Body Weight, kg	53.0 (0.6)	59.8 (0.5)	59.4 (0.8)
Height, cm	161.5 (0.5)	165.0 (0.5)	162.7 (0.8)
BMI	20.3 (0.2)	22.0 (0.1)	22.4 (0.2)
Serum Na, mmol/L	138.9 (0.3)	138.7 (0.1)	139.8 (0.2)
Urine Volume, mL	71.6 (9.8)	76.5 (5.6)	75.1 (11.0)
Urine Osmolality, mmol/kg	561 (45)	459 (22)	382 (32)
Urine Albumin:Creatinine	7.7 (0.6)	6.5 (0.3)	7.7 (0.4)
eGFR, mL/min/1.73 m^2^	128.3 (1.8)	104.7 (1.2)	89.4 (1.4)
Serum Creatinine, mg/dL	0.7 (0.01)	0.7 (0.01)	0.7 (0.01)
HgA1c, %	5.2 (0.03)	5.2 (0.02)	5.5 (0.03)
Fasting HOMA-IR	0.8 (0.05)	0.5 (0.02)	0.5 (0.06)
Fasting Glucose, mg/dL	90.8 (0.7)	89.7 (0.5)	95.6 (0.9)
Fasting Triglyceride, mg/dL	64.0 (2.4)	68.5 (1.5)	83.2 (3.8)
Fasting HDL, mg/dL	62.4 (0.7)	66.0 (1.0)	70.5 (1.9)

Data representative of the US population were obtained from the National Health and Nutrition Examination Surveys (NHANES). The sub-group excluded participants who were older than 70 years, had not fasted for 8 hours, pregnant or lactating women, individuals who self-reported ever having a diagnosis of diabetes or weak or failing kidneys, individuals with a measured body mass index (BMI) outside the normal weight range, and individuals with fasting glycated hemoglobin (HgA1c) ≥ 6.5%, fasting Homeostasis Model Assessment of Insulin Resistance (HOMA-IR) ≥ 2.5, fasting glucose ≥ 126 mg/dL, fasting triglyceride ≥ 150 mg/dL, fasting HDL < 40 mg/dL for men, HDL < 50 mg/dL for women, and/or estimated glomerular filtration rate (eGFR) < 60. Estimates in this table are geometric means and standard errors (SE) with survey weights applied.

**Table 3:** Proportion of the US population and sub-group without selected chronic disease risk factors that met the hydration criteria in 2009–2012 by life-stage group.

	US Population			Sub-Group without Selected Chronic Disease Risk Factors		
	Total	Met Hydration Criteria		Total	Met Hydration Criteria	
		Yes	No		Yes	No
	*n*	Weighted % (95% CI)	Weighted % (95% CI)	*n*	Weighted % (95% CI)	Weighted % (95% CI)
Male						
12–18 years	978	16.5 (11.9–22.5)	83.5 (77.5–88.1)	201	12.8 (8.1–19.5)	87.2 (80.5–91.8)
19–50 years	2703	22.7 (20.1–25.6)	77.3 (74.4–80.0)	234	29.9 (23.6–37.2)	70.1 (62.8–76.4)
51–70 years	1551	27.9 (24.5–31.6)	72.1 (68.4–75.5)	96	43.2 (27.5–60.4)	56.8 (39.6–72.5)
71–80 years	713	28.9 (25.7–32.4)	71.1 (67.6–74.3)			
Female						
12–18 years	882	19.8 (15.7–24.6)	80.2 (75.4–84.3)	132	18.2 (11.5–27.5)	81.8 (72.5–88.5)
19–50 years	2849	28.7 (25.6–31.9)	71.3 (68.1–74.4)	305	32.7 (26.7–39.4)	67.3 (60.6–73.3)
51–70 years	1526	34.1 (29.4–39.0)	65.9 (61.0–70.6)	98	51.0 (36.0–65.8)	49.0 (34.2–64.0)
71–80 years	722	30.8 (26.3–35.7)	69.2 (64.3–73.7)			
Pregnant	108	24.9 (15.3–37.9)	75.1 (62.1–84.7)			

Data representative of the US population were obtained from the National Health and Nutrition Examination Surveys (NHANES). The sub-group excluded participants who were older than 70 years, had not fasted for eight hours, pregnant or lactating women, individuals who self-reported ever having a diagnosis of diabetes or weak or failing kidneys, individuals with a measured BMI outside the normal weight range, and individuals with fasting HgA1c ≥ 6.5%, fasting HOMA-IR ≥ 2.5, fasting glucose ≥ 126 mg/dL, fasting triglyceride ≥ 150 mg/dL, fasting HDL < 40 mg/dL for men, HDL < 50 mg/dL for women, and/or estimated glomerular filtration rate (eGFR) < 60. Participants were classified as hydrated if they had a serum sodium of 135–144 mmol/L, a urine volume ≥ 50 mL, and a urine osmolality < 500 mmol/kg. *n*: Number of NHANES participants in the row category. Weighted % (95% CI): Weighted proportion of the row category who met (or did not meet) the hydration criteria with the corresponding 95% confidence interval.

**Table 4:** Relative risk of meeting the hydration criteria associated with TWI ≥ 45 mL/kg or PWI ≥ 20 mL/kg for the US population aged 12–80 years in 2009–2012.

	Water Intake	Total	Percent Hydrated	Relative Risk of Meeting the Hydration Criteria					
				Unadjusted		Multivariable Adjusted			
				Model 1		Model 2		Model 3	
	TWI ≥ 45 mL/kg or PWI ≥ 20 mL/kg	*n*	Weighted % (95% CI)	Weighted RR (95% CI)	*p*	Weighted RR (95% CI)	*p*	Weighted RR (95% CI)	*p*
US Population									
Male									
12–18 years	No	683	12.8 (9.5–17.1)	1.0		1.0		1.0	
	Yes	295	24.6 (14.6–38.3)	1.92 (1.16–3.19))	0.013	1.77 (1.21–2.59)	0.005	1.58 (1.09–2.30)	0.018
19–50 years	No	1623	19.2 (16.2–22.6)	1.0		1.0		1.0	
	Yes	1080	27.6 (23.6–31.9)	1.43 (1.16–1.77)	0.001	1.50 (1.22–1.84)	0.000	1.36 (1.10–1.68)	0.006
51–70 years	No	1123	21.8 (18.5–25.5)	1.0		1.0		1.0	
	Yes	428	41.7 (33.9–49.9)	1.91 (1.49–2.46)	0.000	1.96 (1.52–2.53)	0.000	1.76 (1.36–2.28)	0.000
71–80 years	No	583	26.3 (22.4–30.7)	1.0		1.0		1.0	
	Yes	130	40.8 (29.8–52.9)	1.55 (1.06–2.26)	0.02	1.48 (1.01–2.17)	0.045	1.47 (0.96–2.24)	0.074
Female									
12–18 years	No	664	15.4 (11.5–20.3)	1.0		1.0		1.0	
	Yes	218	30.0 (22.0–39.4)	1.94 (1.35–2.79)	0.001	1.85 (1.25–2.74)	0.003	1.75 (1.19–2.58)	0.006
19–50 years	No	1759	21.2 (18.1–24.6)	1.0		1.0		1.0	
	Yes	1090	39.8 (35.5–44.3)	1.88 (1.63–2.17)	0.000	1.79 (1.56–2.05)	0.000	1.70 (1.49–1.95)	0.000
51–70 years	No	1038	29.4 (24.4–35.0)	1.0		1.0		1.0	
	Yes	488	42.4 (34.5–50.6)	1.44 (1.12–1.86)	0.006	1.42 (1.07–1.88)	0.016	1.29 (1.00–1.66)	0.051
71–80 years	No	570	29.3 (24.1–35.0)	1.0		1.0		1.0	
	Yes	152	36.2 (27.6–45.8)	1.24 (0.91–1.69)	0.172	1.28 (0.93–1.77)	0.128	1.22 (0.87–1.71)	0.234
Pregnant	No	62	15.3 (7.3–29.4)	1.0		1.0		1.0	
	Yes	46	38.2 (22.7–56.5)	2.50 (1.10–5.66)	0.030	2.08 (0.87–4.93)	0.095	1.78 (0.81–3.90)	0.145
Sub-group without selected chronic disease risk factors									
Male									
12–18 years	No	122	9.7 (5.4–16.6)	1.0		1.0		1.0	
	Yes	79	17.3 (8.7–31.3)	1.79 (0.79–4.05)	0.155	1.99 (0.87–4.54)	0.098	1.83 (0.78–4.29)	0.156
19–50 years	No	129	30.4 (21.4–41.1)	1.0		1.0		1.0	
	Yes	105	29.4 (21.2–39.2)	0.97 (0.62–1.51)	0.885	1.18 (0.69–2.03)	0.536	1.16 (0.67–1.99)	0.587
51–70 years	No	65	30.8 (17.7–48.0)	1.0		1.0		1.0	
	Yes	31	62.7 (34.3–84.4)	2.04 (1.09–3.82)	0.028	1.92 (1.07–3.46)	0.030	2.20 (1.15–4.18)	0.018
Female									
12–18 years	No	84	14.1 (5.5–31.5)	1.0		1.0		1.0	
	Yes	48	23.9 (14.5–36.7)	1.70 (0.57–5.07)	0.333	1.97 (0.66–5.84)	0.214	3.36 (1.23–9.21)	0.020
19–50 years	No	158	24.4 (16.1–35.0)	1.0		1.0		1.0	
	Yes	147	41.2 (35.5–47.2)	1.69 (1.15–2.50)	0.010	1.56 (1.04–2.35)	0.032	1.56 (1.05–2.34)	0.031
51–70 years	No	47	39.7 (21.4–61.6)	1.0		1.0		1.0	
	Yes	51	61.9 (43.4–77.5)	1.56 (0.90–2.71)	0.112	1.88 (1.12–3.17)	0.019	2.00 (1.18–3.40)	0.011

Data representative of the US population were obtained from the 2009–2012 National Health and Nutrition Examination Surveys (NHANES). The sub-group excluded participants who were older than 70 years, had not fasted for eight hours, pregnant or lactating women, individuals who self-reported ever having a diagnosis of diabetes or weak or failing kidneys, individuals with a measured BMI outside the normal weight range, and individuals with fasting HgA1c ≥ 6.5%, fasting HOMA-IR ≥ 2.5, fasting glucose ≥ 126 mg/dL, fasting triglyceride ≥ 150 mg/dL, fasting HDL < 40 mg/dL for men, HDL < 50 mg/dL for women, and/or estimated glomerular filtration rate (eGFR) < 60. Participants were classified as hydrated if they had a serum sodium of 135–144 mmol/L, a urine volume ≥ 50 mL, and a urine osmolality < 500 mmol/kg. *n*, Number of NHANES participants in the row category. Weighted % (95%CI), Weighted proportion of the row category who met the hydration criteria with corresponding 95% confidence interval. The relative risk (RR) of meeting the hydration criteria was estimated using Poisson models with robust standard errors and survey weights applied to all models. Model 1 estimated the RR of meeting the hydration criteria associated with having a total water intake (TWI) ≥ 45 mL/kg or plain water intake (PWI) ≥ 20 mL/kg without adjusting for covariates. Model 2 adjusted the RR estimate for age in years, race–ethnicity, level of physical activity METs, dietary solute load, cigarette smoking, prescription medication use, winter season, fasting status, and morning, afternoon or evening timing of the MEC visit. Model 3 added control for body weight. p, p-value.

**Table 5:** Relative risk of meeting the hydration criteria associated with TWI ≥ 45 mL/kg and PWI ≥ 20 mL/kg for the US population aged 12–80 years in 2009–2012.

		Water Intake	Total	Percent Hydrated	Relative Risk of Meeting Hydration Criteria					
					Unadjusted		Multivariable Adjusted			
					Model 1		Model 2		Model 3	
		mL/kg	*n*	Weighted % (95% CI)	Weighted RR (95% CI)	*p*	Weighted RR (95% CI)	*p*	Weighted RR (95% CI)	*p*
US Population										
Male	12–18 years	TWI < 45 PWI < 20	683	12.8 (9.4–17.1)	1.0		1.0		1.0	
		TWI < 45 PWI ≥ 20	67	8.7 (5.3–13.9)	0.68 (0.40–1.15)	0.145	0.61 (0.33–1.14)	0.118	0.62 (0.32–1.17)	0.135
		TWI ≥ 45 PWI < 20	93	37.5 (17.3–63.2)	2.92 (1.51–5.66)	0.002	2.83 (1.64–4.88)	0.000	2.48 (1.39–4.42)	0.003
		TWI ≥ 45 PWI ≥ 20	135	18.7 (10.2–31.6)	1.46 (0.75–2.81)	0.253	1.45 (0.72–2.90)	0.285	1.33 (0.67–2.61)	0.408
	19–50 years	TWI < 45 PWI < 20	1623	19.2 (16.2–22.6)	1.0		1.0		1.0	
		TWI < 45 PWI ≥ 20	185	19.6 (13.6–27.4)	1.02 (0.69–1.51)	0.926	0.97 (0.66–1.43)	0.886	0.97 (0.66–1.42)	0.867
		TWI ≥ 45 PWI < 20	379	26.6 (18.7–36.2)	1.38 (0.98–1.94)	0.062	1.50 (1.07–2.10)	0.020	1.29 (0.92–1.81)	0.129
		TWI ≥ 45 PWI ≥ 20	516	31.3 (25.7–37.6)	1.63 (1.27–2.09)	0.000	1.75 (1.38–2.22)	0.000	1.58 (1.25–2.01)	0.000
	51–70 years	TWI < 45 PWI < 20	1123	21.8 (18.5–25.5)	1.0		1.0		1.0	
		TWI < 45 PWI ≥ 20	88	25.7 (15.3–39.9)	1.18 (0.69–2.01)	0.532	1.18 (0.69–1.99)	0.534	1.23 (0.74–2.03)	0.414
		TWI ≥ 45 PWI < 20	170	47.5 (33.2–62.3)	2.18 (1.55–3.07)	0.000	2.31 (1.57–3.40)	0.000	1.98 (1.35–2.90)	0.001
		TWI ≥ 45 PWI ≥ 20	170	41.5 (31.3–52.3)	1.90 (1.39–2.60)	0.000	1.99 (1.48–2.69)	0.000	1.80 (1.31–2.48)	0.001
	71–80 years	TWI < 45 PWI < 20	583	26.3 (22.4–30.7)	1.0		1.0		1.0	
		TWI < 45 PWI ≥ 20	39	32.5 (14.6–57.6)	1.23 (0.57–2.65)	0.579	1.20 (0.61–2.37)	0.580	1.21 (0.62–2.34)	0.564
		TWI ≥ 45 PWI < 20	35	38.4 (22.0–58.0)	1.46 (0.83–2.56)	0.180	1.36 (0.78–2.35)	0.269	1.34 (0.73–2.47)	0.332
		TWI ≥ 45 PWI ≥ 20	56	49.1 (34.7–63.7)	1.87 (1.33–2.63)	0.001	1.80 (1.22–2.67)	0.004	1.79 (1.15–2.79)	0.012
Female	12–18 years	TWI < 45 PWI < 20	664	15.4 (11.5–20.3)	1.0		1.0		1.0	
		TWI < 45 PWI ≥20	55	20.6 (10.4–36.8)	1.34 (0.66–2.71)	0.408	1.31 (0.61–2.80)	0.480	1.34 (0.63–2.84)	0.431
		TWI ≥45 PWI < 20	53	28.3 (13.6–50.0)	1.83 (0.85–3.93)	0.116	1.85 (0.86–3.97)	0.114	1.71 (0.75–3.89)	0.191
		TWI ≥45 PWI ≥20	110	34.7 (21.3–51.1)	2.25 (1.46–3.48)	0.001	2.12 (1.27–3.53)	0.005	1.99 (1.17–3.37)	0.012
	19–50 years	TWI < 45 PWI < 20	1759	21.2 (18.1–24.6)	1.0		1.0		1.0	
		TWI < 45 PWI ≥20	290	28.5 (21.9–36.3)	1.35 (1.06–1.71)	0.016	1.35 (1.06–1.71)	0.016	1.36 (1.06–1.73)	0.016
		TWI ≥45 PWI < 20	237	43.7 (35.2–52.4)	2.06 (1.67–2.55)	0.000	2.04 (1.62–2.57)	0.000	1.91 (1.51–2.41)	0.000
		TWI ≥45 PWI ≥20	563	42.8 (37.0–48.7)	2.03 (1.70–2.41)	0.000	1.88 (1.59–2.22)	0.000	1.79 (1.51–2.12)	0.000
	51–70 years	TWI < 45 PWI < 20	1038	29.4 (24.4–35.0)	1.0		1.0		1.0	
		TWI < 45 PWI ≥20	128	33.3 (21.5–47.7)	1.13 (0.72–1.78)	0.585	1.16 (0.74–1.84)	0.487	1.14 (0.72–1.78)	0.569
		TWI ≥45 PWI < 20	131	34.2 (24.5–45.4)	1.16 (0.83–1.62)	0.363	1.13 (0.77–1.65)	0.513	1.01 (0.70–1.45)	0.957
		TWI ≥45 PWI ≥20	229	50.9 (39.9–61.7)	1.73 (1.32–2.27)	0.000	1.69 (1.27–2.27)	0.001	1.52 (1.17–1.98)	0.003
	71–80 years	TWI < 45 PWI < 20	570	29.3 (24.1–35.0)	1.0		1.0		1.0	
		TWI < 45 PWI ≥20	41	31.2 (19.0–46.6)	1.07 (0.64–1.77)	0.802	1.07 (0.62–1.83)	0.813	1.05 (0.61–1.80)	0.849
		TWI ≥45 PWI < 20	41	47.3 (29.1–66.3)	1.62 (1.03–2.53)	0.036	1.67 (1.00–2.79)	0.050	1.56 (0.88–2.75)	0.121
		TWI ≥45 PWI ≥20	70	32.5 (23.5–43.1)	1.11 (0.80–1.54)	0.514	1.20 (0.84–1.72)	0.311	1.14 (0.80–1.63)	0.448
	Pregnant	TWI < 45 PWI < 20	62	15.3 (7.3–29.4)	1.0		1.0		1.0	
		TWI < 45 PWI ≥20	12	66.5 (32.2–89.2)	4.34 (1.85–10.18)	0.001	3.11 (1.13–8.57)	0.030	3.22 (1.13–9.22)	0.030
		TWI ≥45 PWI < 20	7	-	-	-	-	-	-	-
		TWI ≥45 PWI ≥20	27	32.2 (17.1–52.3)	2.11 (0.83–5.34)	0.112	1.95 (0.72–5.29)	0.183	1.53 (0.61–3.81)	0.350
Sub-group without selected chronic disease risk factors										
Male	12–18 years	TWI < 45 PWI < 20	122	9.7 (5.4–16.6)	1.0		1.0		1.0	
		TWI < 45 PWI ≥20	13	20.6 (6.0–51.4)	2.13 (0.74–6.16)	0.156	1.59 (0.70–3.63)	0.262	1.64 (0.71–3.82)	0.241
		TWI ≥ 45 PWI < 20	26	20.0 (7.7–43.0)	2.08 (0.80–5.42)	0.130	2.21 (0.67–7.29)	0.185	1.83 (0.56–6.04)	0.310
		TWI ≥ 45 PWI ≥20	40	14.6 (4.07–37.5)	1.52 (0.42–5.44)	0.512	2.09 (0.61–7.13)	0.230	1.96 (0.55–6.91)	0.287
	19–50 years	TWI < 45 PWI < 20	129	30.4 (21.4–41.1)	1.0		1.0		1.0	
		TWI < 45 PWI ≥20	14	26.0 (6.9–62.5)	0.85 (0.25–2.97)	0.799	1.08 (0.28–4.18)	0.914	1.09 (0.27–4.33)	0.903
		TWI ≥45 PWI < 20	43	29.9 (14.3–52.2)	0.99 (0.50–1.95)	0.965	1.29 (0.62–2.67)	0.489	1.26 (0.60–2.65)	0.538
		TWI ≥ 45 PWI ≥ 20	48	30.3 (17.5–47.2)	1.00 (0.54–1.85)	0.995	1.14 (0.57–2.28)	0.710	1.11 (0.56–2.17)	0.763
	51–70 years	TWI < 45 PWI < 20	65	30.8 (17.7–48.0)	1.0		1.0		1.0	
		TWI < 45 PWI ≥20	5	80.7 (34.5–97.1)	2.62 (1.30–5.27)	0.008	3.21 (1.20–8.54)	0.021	3.49 (1.11–11.0)	0.034
		TWI ≥ 45 PWI < 20	10	44.4 (11.5–83.1)	1.44 (0.50–4.19)	0.489	0.98 (0.43–2.27)	0.968	1.12 (0.45–2.78)	0.797
		TWI ≥45 PWI ≥20	16	75.9 (41.0–93.4)	2.46 (1.34–4.52)	0.005	3.91 (1.59–9.62)	0.004	4.21 (1.64–10.77)	0.004
Female	12–18 years	TWI < 45 PWI < 20	84	14.1 (5.5–31.5)	1.0		1.0		1.0	
		TWI < 45 PWI ≥20	11	12.1 (2.0–48.3)	0.86 (0.10–7.14)	0.888	1.55 (0.20–12.10)	0.666	1.71 (0.21–14.18)	0.607
		TWI ≥45 PWI < 20	12	7.3 (2.3–21.1)	0.52 (0.11–2.37)	0.386	0.37 (0.09–1.51)	0.161	0.71 (0.19–2.62)	0.593
		TWI ≥45 PWI ≥20	25	36.6 (19.9–57.4)	2.61 (0.92–7.38)	0.070	4.40 (1.99–9.70)	0.001	7.49 (2.72–20.62)	0.000
	19–50 years	TWI < 45 PWI < 20	158	24.4 (16.1–35.0)	1.0		1.0		1.0	
		TWI < 45 PWI ≥20	21	27.3 (11.9–51.1)	1.12 (0.54–2.34)	0.757	1.08 (0.53–2.19)	0.830	1.08 (0.53–2.21)	0.833
		TWI ≥45 PWI < 20	38	37.3 (21.5–56.3)	1.53 (0.83–2.81)	0.163	1.38 (0.75–2.53)	0.284	1.38 (0.77–2.50)	0.272
		TWI ≥45 PWI ≥20	88	44.9 (37.1–52.9)	1.84 (1.21–2.82)	0.006	1.71 (1.10–2.68)	0.020	1.71 (1.10–2.68)	0.020
	51–70 years	TWI < 45 PWI < 20	47	39.7 (21.3–61.6)	1.0		1.0		1.0	
		TWI < 45 PWI ≥20	4	64.7 (13.5–95.5)	1.63 (0.60–4.42)	0.328	2.30 (0.84–6.32)	0.102	2.03 (0.68–6.00)	0.194
		TWI ≥45 PWI < 20	14	21.9 (3.9–66.0)	0.55 (0.11–2.85)	0.466	0.78 (0.17–3.59)	0.738	0.80 (0.18–3.63)	0.767
		TWI ≥45 PWI ≥20	33	71.7 (48.9–87.0)	1.80 (1.03–3.17)	0.041	2.07 (1.19–3.60)	0.011	2.27 (1.34–3.86)	0.004

Data representative of the US population were obtained from the 2009–2012 National Health and Nutrition Examination Surveys (NHANES). The sub-group excluded participants who were older than 70 years, had not fasted for eight hours, pregnant or lactating women, individuals who self-reported ever having a diagnosis of diabetes or weak or failing kidneys, individuals with a measured BMI outside the normal weight range, and individuals with fasting HgA1c ≥6.5%, fasting HOMA-IR ≥2.5, fasting glucose ≥126 mg/dL, fasting triglyceride ≥150 mg/dL, fasting HDL < 40 mg/dL for men, HDL < 50 mg/dL for women, and/or estimated glomerular filtration rate (eGFR) < 60. Participants were classified as hydrated if they had a serum sodium of 135–144 mmol/L, a urine volume ≥ 50 mL, and a urine osmolality < 500 mmol/kg. *n*, Number of NHANES participants in the row category. Weighted % (95%CI), Weighted proportion of the row category who met the hydration criteria with corresponding 95% confidence interval. The relative risk (RR) of meeting the hydration criteria was estimated using Poisson models with robust standard errors and survey weights applied to all models. Model 1 estimated the RR of meeting the hydration criteria without adjusting for covariates. Model 2 adjusted the RR estimate for age in years, race–ethnicity, level of physical activity METs, dietary solute load, cigarette smoking, prescription medication use, winter season, fasting status, and morning, afternoon or evening timing of the MEC visit. Model 3 added control for body weight. p, *p*-value.

**Table 6:** Mean difference in TWI associated with meeting the hydration criteria versus not meeting the hydration criteria for the US population aged 12–80 years and the sub-group aged 12–70 years without selected chronic disease risk factors in 2009–2012.

	US Population							Sub-Group without Selected Chronic Disease Risk Factors						
	OLS Regression Coefficients						Predicted Difference in TWI mL/kg	OLS Regression Coefficients						Predicted Difference in TWI mL/kg
	Unadjusted		Multivariable Adjusted					Unadjusted		Multivariable Adjusted				
	Model 1		Model 2		Model 3			Model 1		Model 2		Model 3		
	B (SE)	*p*	B (SE)	*p*	B (SE)	*p*	Mean (SE)	B (SE)	*p*	B (SE)	*p*	B (SE)	*p*	Mean (SE)
Male														
12–18 years	11.2 (4.6)	0.020	8.9 (2.5)	0.001	7.1 (2.3)	0.003	7.1 (2.3)	8.8 (5.2)	0.099	9.5 (4.5)	0.042	8.2 (4.4)	0.069	8.2 (4.4)
19–50 years	5.0 (1.4)	0.001	5.5 (1.2)	0.000	3.5 (1.2)	0.005	3.5 (1.2)	2.7 (3.7)	0.474	4.9 (3.1)	0.130	4.3 (3.0)	0.159	4.3 (3.0)
51–70 years	8.1 (1.8)	0.000	7.7 (1.2)	0.000	5.7 (1.3)	0.000	5.7 (1.3)	11.5 (9.0)	0.212	10.4 (6.6)	0.127	11.3 (6.6)	0.098	11.3 (6.6)
71–80 years	5.6 (1.4)	0.000	4.6 (1.4)	0.002	4.4 (1.4)	0.005	4.4 (1.4)	-	-	-	-	-	-	-
Female														
12–18 years	6.2 (2.6)	0.024	5.2 (2.2)	0.024	3.9 (2.0)	0.056	3.9 (2.0)	11.9 (5.3)	0.033	11.2 (5.5)	0.050	14.3 (5.1)	0.009	14.3 (5.1)
19–50 years	10.7 (1.2)	0.000	9.6 (1.1)	0.000	8.1 (0.9)	0.000	8.1 (0.9)	9.0 (3.1)	0.006	7.2 (3.3)	0.036	7.1 (3.2)	0.031	7.1 (3.2)
51–70 years	7.4 (2.1)	0.001	7.1 (2.2)	0.003	5.2 (1.9)	0.009	5.2 (1.9)	7.9 (5.6)	0.167	11.4 (5.0)	0.030	11.8 (4.8)	0.019	11.8 (4.8)
71–80 years	2.8 (1.5)	0.064	3.5 (1.3)	0.012	2.8 (1.2)	0.029	2.8 (1.2)	-	-	-	-	-	-	-
Pregnant	7.6 (4.1)	0.075	5.0 (3.4)	0.151	1.0 (3.3)	0.764	1.0 (3.3)	-	-	-	-	-	-	-

Data representative of the US population were obtained from the 2009–2012 National Health and Nutrition Examination Surveys (NHANES). The study population included all participants aged 12–80 years with data on water intake, serum sodium, and body weight. The sub-group excluded participants who were older than 70 years, had not fasted for 8 hours, pregnant or lactating women, individuals who self-reported ever having a diagnosis of diabetes or weak or failing kidneys, individuals with a measured BMI outside the normal weight range, and individuals with fasting HgA1c ≥ 6.5%, fasting HOMA-IR ≥ 2.5, fasting glucose ≥ 126 mg/dL, fasting triglyceride ≥ 150 mg/dL, fasting HDL < 40 mg/dL for men, HDL < 50 mg/dL for women, and/or estimated glomerular filtration rate (eGFR) < 60. Participants were classified as hydrated if they had a serum sodium of 135–144 mmol/L, a urine volume ≥ 50 mL, and a urine osmolality < 500 mmol/kg. B (SE), regression coefficient and corresponding standard error. The regression coefficients represent sex- and age-specific mean differences in total water intake (TWI) associated with meeting versus not meeting the hydration criteria, estimated by linear regression (OLS) models. Model 1 estimated the difference in TWI without adjusting for covariates. Model 2 adjusted for age in years, race–ethnicity, level of physical activity METs, dietary solute load, cigarette smoking, prescription medication use, winter season, timing of the MEC visit, and fasting status. Model 3 added control for body weight. Sex- and age-specific mean differences in TWI were predicted from Model 3, holding all covariates at their mean values. p, *p*-value.

**Table 7:** Mean difference in PWI associated with meeting the hydration criteria versus not meeting the hydration criteria for the US population aged 12–80 years and the sub-group aged 12–70 years without selected chronic disease risk factors in 2009–2012.

	US Population							Sub-group without Selected Chronic Disease Risk Factors						
	ZINB Model Coefficients						Predicted Difference in PWI mL/kg	ZINB Model Coefficients						Predicted Difference in PWI mL/kg
	Unadjusted		Multivariable Adjusted					Unadjusted		Multivariable Adjusted				
	Model 1		Model 2		Model 3			Model 1		Model 2		Model 3		
	B (SE)	*p*	B (SE)	*p*	B (SE)	*p*	Mean (SE)	B (SE)	*p*	B (SE)	*p*	B (SE)	*p*	Mean (SE)
Male														
12–18 years	−0.01 (0.19)	0.979	0.01 (0.18)	0.946	−0.02 (0.18)	0.917	−0.2 (2.1)	0.23 (0.25)	0.368	0.40 (0.24)	0.101	0.41 (0.24)	0.103	7.1 (4.6)
19–50 years	0.15 (0.06)	0.020	0.16 (0.06)	0.009	0.12 (0.06)	0.035	1.9 (0.9)	0.03 (0.13)	0.815	0.12 (0.15)	0.441	0.09 (0.15)	0.548	1.4 (2.3)
51–70 years	0.24 (0.09)	0.012	0.21 (0.08)	0.015	0.16 (0.09)	0.069	1.8 (1.0)	0.78 (0.29)	0.011	0.72 (0.24)	0.005	0.73 (0.25)	0.006	8.1 (3.0)
71–80 years	0.22 (0.10)	0.040	0.19 (0.11)	0.102	0.18 (0.11)	0.107	1.6 (1.0)	-	-	-	-	-	-	-
Female														
12–18 years	0.23 (0.09)	0.011	0.22 (0.11)	0.054	0.16 (0.09)	0.081	2.1 (1.2)	0.41 (0.26)	0.131	0.42 (0.26)	0.113	0.52 (0.19)	0.011	8.7 (3.8)
19–50 years	0.29 (0.05)	0.000	0.27 (0.05)	0.000	0.23 (0.04)	0.000	3.8 (0.7)	0.21 (0.14)	0.144	0.19 (0.15)	0.202	0.20 (0.15)	0.188	3.9 (3.0)
51–70 years	0.24 (0.09)	0.010	0.21 (0.08)	0.016	0.15 (0.07)	0.034	2.0 (0.9)	0.40 (0.18)	0.035	0.53 (0.18)	0.007	0.51 (0.19)	0.010	9.6 (4.2)
71–80 years	0.02 (0.08)	0.849	0.02 (0.07)	0.777	0.00 (0.08)	0.993	0.0 (0.9)	-	-	-	-	-	-	-
Pregnant	0.39 (0.17)	0.028	0.49 (0.18)	0.010	0.49 (0.21)	0.026	10.0 (4.7)	-	-	-	-	-	-	-

Data representative of the US population were obtained from the 2009–2012 National Health and Nutrition Examination Surveys (NHANES). The study population included all participants aged 12–80 years with data on water intake, serum sodium, and body weight. The sub-group excluded participants who were older than 70 year, had not fasted for 8 hour, pregnant or lactating women, individuals who self-reported ever having a diagnosis of diabetes or weak or failing kidneys, individuals with a measured BMI outside the normal weight range, and individuals with fasting HgA1c ≥ 6.5%, fasting HOMA-IR ≥ 2.5, fasting glucose ≥ 126 mg/dL, fasting triglyceride ≥ 150 mg/dL, fasting HDL < 40 mg/dL for men, HDL < 50 mg/dL for women, and/or estimated glomerular filtration rate (eGFR) < 60. Participants were classified as hydrated if they had a serum sodium of 135–144 mmol/L, a urine volume ≥ 50 mL, and a urine osmolality < 500 mmol/kg. B (SE), regression coefficient and corresponding standard error. The regression coefficients represent the log difference in plain water intake (PWI) associated with meeting versus not meeting the hydration criteria, estimated by survey weighted zero-inflated negative binomial (ZINB) regression models. Model 1 estimated the difference in PWI without adjusting for covariates. Model 2 adjusted for age in years, race–ethnicity, level of physical activity METs, dietary solute load, cigarette smoking, prescription medication use, winter season, timing of the MEC visit, and fasting status. Model 3 added control for body weight. Sex- and age-specific mean differences in PWI were predicted from Model 3, holding all covariates at their mean values. p, *p*-value.

**Table 8:** Percentiles of the TWI and PWI distributions of the US population aged 12–80 years in 2009–2012 by hydration classification and life-stage group.

			Percentiles of the TWI and PWI Distributions									
			Did Not Meet Hydration Criteria					Met Hydration Criteria				
			10	25	50	75	90	10	25	50	75	90
			Weighted Estimate (SE)					Weighted Estimate (SE)				
US Population												
Male	12–18 years	TWI, L/d	1.2 (0.1)	1.6 (0.1)	2.2 (0.1)	2.9 (0.1)	3.9 (0.2)	1.3 (0.1)	1.8 (0.1)	2.8 (0.3)	3.5 (0.5)	5.1 (0.4)
		PWI, L/d	0	0.1 (0.1)	0.5 (0.04)	1.2 (0.1)	1.9 (0.2)	0	0	0.4 (0.2)	1.0 (0.4)	2.3 (0.6)
		TWI, mL/kg	17.5 (0.8)	23.9 (0.9)	32.4 (1.3)	42.7 (2.5)	58.2 (2.5)	21.3 (2.7)	31.2 (2.1)	41.5 (3.4)	57.1 (4.7)	74.7 (15.1)
		PWI, mL/kg	0	1.9 (0.9)	8.5 (0.7)	17.6 (1.6)	28.6 (2.6)	0	0	6.6 (2.2)	17.5 (6.6)	34.6 (7.8)
	19–50 years	TWI, L/d	1.8 (0.05)	2.4 (0.1)	3.2 (0.1)	4.3 (0.1)	5.8 (0.2)	1.9 (0.1)	2.7 (0.1)	3.5 (0.1)	4.5 (0.1)	5.6 (0.2)
		PWI, L/d	0	0.2 (0.1)	0.9 (0.1)	1.9 (0.1)	3.0 (0.1)	0	0.4 (0.1)	1.1 (0.1)	2.1 (0.1)	3.2 (0.1)
		TWI, mL/kg	20.2 (0.6)	27.0 (0.6)	36.6 (0.8)	48.8 (1.4)	67.0 (2.5)	23.3 (0.9)	31.7 (1.4)	41.9 (1.6)	55.6 (1.9)	73.4 (2.9)
		PWI, mL/kg	0	2.7 (0.9)	10.0 (0.6)	20.8 (1.1)	33.9 (1.2)	0	4.4 (0.7)	13.7 (1.1)	25.4 (0.8)	39.9 (2.5)
	51–70 years	TWI, L/d	1.7 (0.1)	2.3 (0.1)	2.9 (0.1)	4.0 (0.2)	5.0 (0.2)	1.9 (0.1)	2.6 (0.1)	3.4 (0.1)	4.3 (0.3)	5.6 (0.5)
		PWI, L/d	0	0.1 (0.05)	0.6 (0.1)	1.4 (0.1)	2.1 (0.2)	0	0.1 (0.1)	0.7 (0.1)	1.6 (0.1)	2.6 (0.4)
		TWI, mL/kg	18.2 (0.7)	24.6 (0.6)	32.8 (1.1)	42.3 (1.2)	55.5 (2.1)	22.9 (1.1)	29.1 (1.4)	41.5 (1.4)	51.1 (2.4)	67.8 (6.2)
		PWI, mL/kg	0	0.7 (0.5)	6.6 (0.7)	15.4 (1.1)	24.6 (1.1)	0	1.5 (0.7)	8.5 (1.3)	19.3 (1.9)	30.3 (5.4)
	71–80 years	TWI, L/d	1.3 (0.04)	1.7 (0.05)	2.2 (0.1)	3.0 (0.1)	3.7 (0.2)	1.7 (0.1)	1.9 (0.1)	2.5 (0.1)	3.3 (0.2)	4.2 (0.2)
		PWI, L/d	0	0.01 (0.03)	0.5 (0.04)	0.9 (0.04)	1.7 (0.1)	0	0.2 (0.1)	0.6 (0.1)	1.2 (0.1)	1.9 (0.2)
		TWI, mL/kg	15.2 (40.8)	20.0 (0.6)	26.7 (0.9)	35.7 (0.9)	45.1 (1.7)	18.5 (1.5)	24.5 (1.1)	31.6 (1.5)	42.2 (2.2)	52.3 (4.7)
		PWI, mL/kg	0	0.2 (0.4)	5.2 (0.5)	11.1 (0.9)	19.7 (1.0)	0	2.4 (0.9)	7.2 (1.1)	13.4 (1.8)	24.2 (1.9)
Female	12–18 years	TWI, L/d	0.9 (0.04)	1.3 (0.1)	1.8 (0.1)	2.4 (0.1)	3.0 (0.1)	1.0 (0.1)	1.3 (0.1)	2.1 (0.2)	2.9 (0.3)	3.7 (0.6)
		PWI, L/d	0	0.1 (0.1)	0.5 (0.01)	1.0 (0.1)	1.8 (0.2)	0	0.2 (0.1)	0.7 (0.1)	1.4 (0.2)	2.1 (0.3)
		TWI, mL/kg	14.8 (0.6)	20.0 (1.0)	30.3 (1.3)	42.8 (2.0)	51.2 (3.1)	17.2 (1.5)	22.9 (2.6)	34.9 (5.3)	53.2 (4.8)	60.9 (3.6)
		PWI, mL/kg	0	1.5 (0.8)	9.2 (0.5)	16.2 (1.7)	29.0 (3.9)	0	2.7 (2.0)	11.0 (2.5)	27.1 (3.3)	32.2 (1.8)
	19–50 years	TWI, L/d	1.4 (0.03)	1.8 (0.03)	2.5 (0.04)	3.3 (0.1)	4.2 (0.1)	1.7 (0.1)	2.2 (0.1)	3.0 (0.1)	4.0 (0.1)	5.2 (0.2)
		PWI, L/d	0	0.2 (0.04)	0.8 (0.04)	1.6 (0.1)	2.5 (0.1)	0	0.5 (0.1)	1.2 (0.1)	2.0 (0.2)	3.0 (0.2)
		TWI, mL/kg	17.5 (0.4)	23.2 (0.6)	33.2 (0.7)	44.9 (0.8)	58.4 (1.1)	23.3 (1.0)	30.4 (1.1)	42.5 (1.5)	60.5 (2.5)	78.7 (3.0)
		PWI, mL/kg	0	3.0 (0.5)	10.2 (0.6)	21.5 (1.1)	32.9 (1.3)	0	6.7 (0.7)	16.2 (1.3)	29.6 (1.7)	45.6 (2.4)
	51–70 years	TWI, L/d	1.4 (0.1)	1.9 (0.03)	2.5 (0.04)	3.4 (0.1)	4.3 (0.1)	1.6 (0.1)	2.1 (0.1)	2.7 (0.1)	3.6 (0.2)	4.6 (0.3)
		PWI, L/d	0	0.2 (0.05)	0.7 (0.1)	1.4 (0.1)	2.1 (0.1)	0	0.3 (0.1)	0.9 (0.1)	1.7 (0.1)	2.2 (0.2)
		TWI, mL/kg	18.4 (0.6)	23.4 (0.4)	32.3 (0.6)	44.7 (1.4)	57.4 (2.7)	20.3 (1.6)	29.0 (1.2)	39.8 (1.7)	52.4 (2.5)	70.3 (7.4)
		PWI, mL/kg	0	2.9 (0.5)	10.0 (0.7)	17.8 (0.8)	27.3 (1.6)	0	3.9 (0.9)	13.5 (1.9)	23.3 (2.3)	33.7 (2.3)
	71–80 years	TWI, L/d	1.1 (0.03)	1.5 (0.1)	2.1 (0.1)	2.7 (0.1)	3.3 (0.1)	1.3 (0.1)	1.7 (0.1)	2.2 (0.1)	2.8 (0.2)	3.5 (0.2)
		PWI, L/d	0	0.2 (0.05)	0.6 (0.1)	1.1 (0.1)	1.6 (0.1)	0	0.2 (0.1)	0.5 (0.03)	1.0 (0.1)	1.6 (0.1)
		TWI, mL/kg	16.6 (0.9)	21.9 (0.8)	30.1 (0.8)	38.3 (1.2)	48.7 (0.8)	18.1 (1.2)	23.9 (0.9)	32.1 (2.0)	42.2 (2.0)	56.2 (2.1)
		PWI, mL/kg	0	2.9 (0.8)	8.4 (0.8)	15.0 (0.7)	24.3 (1.4)	0	2.9 (1.0)	8.2 (0.6)	14.2 (1.7)	26.0 (2.7)
	Pregnant	TWI, L/d	1.5 (0.2)	2.3 (0.1)	2.8 (0.2)	4.0 (0.2)	4.8 (0.3)	2.3 (0.3)	2.8 (0.4)	3.2 (0.2)	3.7 (0.7)	4.7 (0.5)
		PWI, L/d	0.1 (0.1)	0.5 (0.1)	1.0 (0.2)	2.3 (0.4)	3.0 (0.6)	0.5 (0.1)	1.0 (0.4)	1.8 (0.4)	2.7 (0.5)	3.6 (0.4)
		TWI, mL/kg	15.3 (3.9)	23.7 (2.4)	37.3 (2.9)	52.7 (3.8)	64.7 (6.3)	23.7 (3.6)	35.4 (5.0)	42.0 (4.6)	53.7 (12.5)	60.5 (5.6)
		PWI, mL/kg	0.9 (0.8)	5.3 (1.5)	10.8 (2.0)	25.8 (5.3)	40.2 (5.7)	5.7 (2.2)	12.6 (4.9)	25.1 (6.4)	38.0 (8.8)	45.6 (3.8)
Sub-group without selected chronic disease risk factors												
Male	12–18 years	TWI, L/d	1.1 (0.1)	1.5 (0.1)	2.1 (0.2)	2.9 (0.1)	3.8 (0.8)	1.6 (0.2)	1.9 (0.3)	2.6 (0.2)	3.0 (0.8)	5.0 (1.0)
		PWI, L/d	0	0.1 (0.1)	0.7 (0.1)	1.3 (0.2)	2.1 (1.2)	0	0	0.6 (0.4)	1.8 (0.9)	2.8 (0.2)
		TWI, mL/kg	20.0 (1.3)	25.8 (1.1)	35.2 (1.7)	50.0 (2.3)	60.6 (8.1)	24.5 (4.4)	34.1 (4.7)	43.9 (6.2)	60.4 (8.4)	79.6 (9.6)
		PWI, mL/kg	0	3.0 (1.6)	11.9 (2.1)	21.7 (2.9)	33.4 (9.5)	0	0	10.0 (6.6)	31.8 (14.1)	42.3 (3.0)
	19–50 years	TWI, L/d	1.5 (0.1)	2.2 (0.2)	2.8 (0.1)	3.6 (0.2)	4.5 (0.6)	1.6 (0.1)	2.1 (0.2)	2.9 (0.2)	4.0 (0.3)	4.9 (0.5)
		PWI, L/d	0	0.1 (0.1)	0.7 (0.1)	1.6 (0.2)	2.2 (0.3)	0	0.4 (0.1)	0.9 (0.1)	1.5 (0.3)	2.4 (0.5)
		TWI, mL/kg	21.8 (2.4)	30.0 (1.8)	40.2 (2.0)	49.3 (2.7)	67.2 (6.9)	23.9 (2.5)	31.9 (2.8)	39.7 (3.6)	51.8 (7.1)	73.9 (19.0)
		PWI, mL/kg	0	1.8 (1.2)	9.4 (1.2)	22.2 (3.0)	35.2 (6.8)	0	6.2 (2.1)	13.6 (1.7)	20.6 (3.8)	33.4 (7.1)
	51–70 years	TWI, L/d	1.8 (0.3)	2.1 (0.1)	2.6 (0.2)	3.1 (0.2)	4.3 (0.4)	1.9 (0.3)	2.3 (0.2)	2.8 (0.4)	3.9 (1.5)	7.7 (1.9)
		PWI, L/d	0	0	0.2 (0.1)	0.6 (0.2)	1.3 (0.3)	0	0.4 (0.2)	1.2 (0.4)	1.9 (0.4)	3.8 (1.0)
		TWI, mL/kg	27.0 (3.9)	31.2 (2.3)	35.9 (3.2)	44.7 (5.5)	61.4 (8.7)	23.7 (4.3)	31.1 (3.5)	41.9 (4.2)	64.0 (13.1)	108.8 (22.4)
		PWI, mL/kg	0	0	3.6 (2.2)	9.3 (2.9)	17.8 (5.6)	0	6.4 (3.6)	14.3 (5.2)	25.4 (5.6)	45.5 (10.2)
Female	12–18 years	TWI, L/d	1.0 (0.1)	1.5 (0.1)	1.9 (0.1)	2.5 (0.3)	3.2 (0.2)	1.1 (0.1)	1.5 (0.4)	2.5 (0.4)	3.7 (0.9)	4.4 (0.3)
		PWI, L/d	0	0	0.5 (0.1)	1.1 (0.1)	1.6 (0.3)	0	0	0.7 (0.5)	1.9 (1.1)	2.8 (0.5)
		TWI, mL/kg	21.2 (1.7)	26.9 (1.6)	34.3 (3.6)	49.5 (5.4)	58.8 (4.8)	23.2 (3.6)	30.7 (6.0)	47.7 (10.9)	65.3 (12.8)	77.7 (6.2)
		PWI, mL/kg	0	0	10.3 (2.2)	19.5 (2.4)	31.3 (6.3)	0	0	16.6 (10.0)	36.7 (21.1)	45.9 (4.6)
	19–50 years	TWI, L/d	1.4 (0.1)	1.8 (0.1)	2.4 (0.1)	3.3 (0.2)	4.2 (0.4)	1.6 (0.2)	2.0 (0.1)	3.0 (0.4)	4.4 (0.2)	4.9 (0.2)
		PWI, L/d	0	0.3 (0.1)	0.8 (0.1)	1.5 (0.1)	2.2 (0.2)	0	0.4 (0.1)	1.2 (0.2)	2.1 (0.2)	3.0 (0.4)
		TWI, mL/kg	23.9 (1.4)	29.5 (1.1)	38.4 (1.9)	54.0 (2.8)	72.1 (4.6)	27.0 (3.1)	33.8 (3.0)	48.3 (6.0)	70.8 (2.9)	82.8 (3.3)
		PWI, mL/kg	0	5.3 (1.5)	12.2 (1.5)	25.3 (2.5)	37.2 (3.2)	0	5.8 (1.9)	19.0 (3.5)	33.7 (3.7)	47.3 (5.4)
	51–70 years	TWI, L/d	1.3 (0.1)	1.8 (0.2)	2.2 (0.2)	3.0 (0.3)	4.3 (0.5)	1.5 (0.2)	1.9 (0.3)	2.7 (0.3)	3.8 (0.5)	4.8 (0.5)
		PWI, L/d	0	0	0.7 (0.2)	1.2 (0.1)	2.1 (0.7)	0	0.4 (0.3)	1.3 (0.4)	2.2 (0.4)	3.1 (0.5)
		TWI, mL/kg	23.7 (3.1)	31.1 (2.3)	37.7 (3.3)	52.1 (4.6)	70.1 (7.3)	23.4 (4.2)	31.8 (5.9)	45.9 (4.8)	68.2 (7.5)	78.2 (5.0)
		PWI, mL/kg	0	0	12.6 (3.4)	20.0 (3.3)	34.9 (10.1)	0	6.5 (5.3)	22.7 (6.0)	37.7 (7.8)	51.5 (6.9)

Data representative of the US population were obtained from the 2009–2012 National Health and Nutrition Examination Surveys (NHANES). The study population included all participants aged 12–80 years with data on water intake, serum sodium, and body weight. The sub-group excluded participants who were older than 70 years, had not fasted for 8 hours, pregnant or lactating women, individuals who self-reported ever having a diagnosis of diabetes or weak or failing kidneys, individuals with a measured BMI outside the normal weight range, and individuals with fasting HgA1c ≥ 6.5%, fasting HOMA-IR ≥ 2.5, fasting glucose ≥ 126 mg/dL, fasting triglyceride ≥ 150 mg/dL, fasting HDL < 40 mg/dL for men, HDL < 50 mg/dL for women, and/or estimated glomerular filtration rate (eGFR) < 60. Participants were classified as hydrated if they had a serum sodium of 135–144 mmol/L, a urine volume ≥ 50 mL, and a urine osmolality < 500 mmol/kg. The percentiles of total water intake (TWI) and plain water intake (PWI) and corresponding standard errors (SE) were estimated with survey weights applied.

## Supplementary Materials

The following are available online at https://public.tableau.com/shared/R47XPPFSK?:toolbar=no&:display_count=no, Figure S1: The dashboard includes the predicted probability of meeting the hydration criteria and corresponding 95% confidence interval for each combination of TWI or PWI for the US population as well as for the sub-group without selected chronic disease risk factors.

## Appendix A

**Table A1:** Determinants of water intake requirements for the US population aged 12–80 years in 2009–2012.

		Age 12–18 years		Age 19–50 years		Age 51–70 years		Age 71–80 years		Pregnant	
		*n*	Weighted % (SE)	*n*	Weighted % (SE)	*n*	Weighted % (SE)	*n*	Weighted % (SE)	*n*	Weighted% (SE)
US Population											
Male		978		2703		1551		713		-	
Body weight, kg	<70	574	56.4 (2.8)	520	15.4 (1.2)	264	11.1 (1.3)	155	19.1 (1.7)	-	-
	70–79	180	17.8 (1.7)	595	21.2 (1.2)	315	17.8 (1.3)	184	25.7 (2.1)	-	-
	80–94	126	16.6 (1.7)	818	33.4 (1.5	498	34.8 (2.6)	232	35.7 (1.9)	-	-
	≥95	98	9.2 (1.3)	770	30.0 (1.2)	474	36.2 (2.3)	142	19.5 (1.7)	-	-
Race–ethnicity	White	283	55.9 (3.9)	1092	62.6 (2.7)	634	76.2 (2.4)	479	81.5 (2.5)	-	-
	Hispanic	336	22.3 (2.9)	746	18.8 (2.4)	399	8.8 (1.6)	81	6.4 (1.4)	-	-
	Black	240	13.2 (1.9)	530	10.9 (1.2)	389	9.4 (1.3)	107	7.4 (1.4)	-	-
	Other	119	8.5 (1.9)	335	7.8 (0.8)	129	5.6 (0.9)	46	4.7 (1.1)	-	-
Activity METs, min/week	<1000	210	19.9 (2.2)	789	25.9 (1.5)	763	42.9 (1.7)	457	61.5 (2.3)	-	-
	1000–4999	492	49.9 (3.2)	939	37.0 (1.6)	470	35.4 (1.6)	192	29.5 (2.3)	-	-
	≥5000	276	30.2 (3.6)	975	37.1 (1.9)	318	21.7 (1.8)	64	9.0 (1.6)	-	-
Dietary solute, mOsm/day	<500	253	24.2 (2.5)	391	13.1 (0.6)	326	15.9 (1.6)	212	24.1 (2.1)	-	-
	500–999	528	52.4 (2.4)	1479	54.4 (1.3)	910	60.0 (1.9)	447	68.5 (1.9)	-	-
	1000–1499	163	19.1 (2.8)	667	25.7 (1.2)	269	20.7 (1.5)	50	7.0 (1.2)	-	-
	≥1500	34	4.3 (0.9)	166	6.8 (0.8)	46	3.4 (0.7)	4	0.4 (0.2)	-	-
Current cigarette smoker	Yes	-	-	742	24.6 (1.6)	369	21.8 (1.8)	66	7.1 (0.9)	-	-
	No	-	-	1810	71.5 (1.3)	1182	78.2 (1.8)	647	92.9 (0.9)	-	-
	Unknown	978	100	151	3.9 (0.7)	0	0	0	0	-	-
Prescription medication	Yes	202	22.5 (1.9)	788	33.8 (1.4)	1072	72.4 (1.6)	641	89.6 (1.5)	-	-
	No	776	77.5 (1.9)	1915	66.2 (1.4)	479	27.6 (1.6)	72	10.4 (1.5)	-	-
MEC visit timing	Winter	482	43.4 (5.7)	1270	44.3 (4.7)	735	37.9 (4.6)	299	42.8 (6.1)	-	-
	Summer	496	56.6 (5.7)	1433	55.7 (4.7)	816	62.1 (4.6)	414	57.2 (6.1)	-	-
8-hour fasted	Yes	462	43.0 (2.9)	1252	42.1 (1.9)	726	45.1 (2.1)	307	43.6 (2.1)	-	-
	No	516	57.0 (2.9)	1451	57.9 (1.9)	825	54.9 (2.1)	406	56.4 (2.1)	-	-
One or more chronic disease risk factor *	Yes	229	48.7 (3.1)	1012	81.1 (1.8)	623	85.4 (1.5)	277	89.4 (2.7)	-	-
	No	233	51.3 (3.1)	240	18.9 (1.8)	101	14.6 (1.5)	28	10.6 (2.7)	-	-
Female		882		2849		1526		722		108	
Body weight, kg	<60	483	57.0 (2.3)	658	22.2 (1.1)	249	18.4 (1.6)	208	27.2 (2.0)	17	12.7 (4.0)
	60–69	184	20.8 (2.2)	659	24.5 (1.0)	318	24.1 (2.4)	196	29.5 (2.1)	23	19.8 (5.7)
	70–84	133	13.7 (1.9)	739	27.0 (1.6)	478	27.7 (1.6)	180	24.4 (1.8)	28	27.8 (4.9)
	≥85	82	8.4 (1.1)	793	26.3 (1.1)	481	29.8 (1.8)	138	18.8 (1.6)	40	39.7 (6.0)
Race-ethnicity	White	243	59.5 (3.3)	1151	60.9 (3.3)	591	74.0 (2.5)	488	82.8 (2.0)	36	55.4 (6.3)
	Hispanic	316	20.3 (2.4)	773	17.4 (2.4)	424	9.5 (1.7)	96	6.2 (1.3)	31	20.8 (5.3)
	Black	207	12.5 (1.8)	604	13.4 (1.6)	391	11.4 (1.6).	105	6.6 (1.0)	27	14.0 (3.1)
	Other	116	7.8 (1.2)	321	8.3 (0.8)	120	5.2 (0.8)	33	4.4 (0.9)	14	9.7 (3.3)
Activity METs, min/wk	<1000	385	38.4 (2.1)	1430	47.4 (1.9)	972	58.5 (2.0)	551	72.2 (2.2)	73	64.9 (6.6)
	1000–4999	390	47.4 (2.4)	985	47.4 (1.9)	405	32.3 (1.7)	140	22.6 (2.1)	24	26.3 (5.7)
	≥5000	107	14.2 (2.3)	434	16.2 (1.1)	149	9.2 (0.9	31	5.2 (1.0)	11	8.8 (4.4)
Dietary solute, mOsm/d	<500	398	46.0 (2.5)	1040	37.1 (1.1)	631	38.4 (1.9)	394	51.5 (2.1)	23	24.1 (6.4)
	500–999	441	50.6 (2.5)	1601	56.9 (1.4)	825	58.1 (1.9)	309	45.7 (1.9)	73	67.6 (5.5)
	1000–1499	40	2.7 (0.8)	194	3.3 (0.6)	67	5.6 (0.7)	17	3.2 (0.8)	11	7.3 (3.2)
	≥1500	3	0.2 (0.1)	14	0.4 (0.1)	3	0.1 (0.0)	2	0.1 (0.0)	1	1.0 (1.0)
Current cigarette smoker	Yes	-	-	599	21.7 (1.4)	226	14.5 (1.4)	40	5.6 (0.9)	15	10.7 (3.0)
	No	-	-	2136	75.5 (1.4)	1300	85.5 (1.4)	682	94.4(0.9)	93	89.3 (3.0)
	Unknown	882	100	114	2.8 (0.5)	0	0	0	0	0	0
Prescription medication	Yes	215	27.9 (2.9)	1300	51.8 (1.9)	1172	76.6 (1.3)	671	92.9 (1.1)	27	29.5 (6.7)
	No	667	72.1 (2.9)	1549	48.2 (1.9)	354	23.4 (1.3)	51	7.1 (1.1)	81	70.5 (6.7)
MEC visit timing	Winter	437	38.6 (5.3)	1463	45.7 (4.6)	811	39.2 (5.1)	412	41.9 (5.5)	47	36.7 (7.4)
	Summer	445	61.4 (5.3)	1386	54.3(4.6)	715	60.8 (5.1)	310	58.1(5.5)	61	63.3 (7.4)
8-hour fasted	Yes	421	44.1 (4.1)	1341	41.4 (1.4)	757	47.5 (1.5)	338	46.6 (2.0)	46	30.8 (5.2)
	No	461	55.9 (4.1)	1508	58.6 (1.4)	769	52.5 (1.5)	384	53.4 (2.0)	62	69.2 (5.2)
One or more chronic disease risk factor *	Yes	242	54.4 (3.7)	988	70.4 (2.1)	643	81.3 (1.6)	288	84.9 (2.2)	37	89.2 (4.2)
	No	179	45.6 (3.7)	353	29.6 (2.1)	114	18.7 (1.6)	50	15.1(2.2)	9	10.8 (4.2)
Sub-group without selected chronic disease risk factors											
Male		201		234		96		-		-	
Body weight, kg	<70	168	84.2 (3.8)	140	51.9 (4.5)	55	41.0 (7.7)	-	-	-	-
	70–79	32	14.8 (3.5)	74	35.8 (4.3)	30	45.4 (6.7)	-	-	-	-
	80–94	1	1.0 (1.0)	20	12.3 (3.0)	11	13.6 (5.5)	-	-	-	-
	≥95	0	0	0	0	0	0	-	-	-	-
Race–ethnicity	White	60	57.1 (4.7)	89	62.3 (4.4)	40	72.4 (4.9)	-	-	-	-
	Hispanic	60	18.9 (3.2)	50	13.1 (2.6)	19	6.4 (1.9)	-	-	-	-
	Black	55	16.7 (2.9)	47	13.7 (2.6)	23	13.1 (3.4)	-	-	-	-
	Other	26	7.3 (1.9)	48	11.0 (2.7)	14	8.1 (3.6)	-	-	-	-
Activity METs, min/wk	<1000	35	15.3 (4.3)	66	28.7 (2.7)	41	30.0 (5.5)	-	-	-	-
	1000–4999	110	56.3 (5.3)	76	29.9 (3.5)	27	37.0 (7.2)	-	-	-	-
	≥5000	56	28.4 (5.2)	92	41.3 (4.0)	28	33.0 (8.3)	-	-	-	-
Dietary solute, mOsm/d	<500	54	29.3 (5.1)	30	14.1 (4.0)	17	15.2 (4.6)	-	-	-	-
	500–999	111	55.8 (4.7)	144	57.1 (4.2)	56	58.9 (6.8)	-	-	-	-
	1000–1499	34	14.3 (2.7)	51	24.7 (4.1)	21	23.6 (5.0)	-	-	-	-
	≥1500	2	0.7 (0.5)	9	4.2 (1.7)	2	2.2 (1.8)	-	-	-	-
Current cigarette smoker	Yes		-	66	26.7 (3.9)	24	29.7 (7.9)	-	-	-	-
	No		-	139	64.6 (3.8)	72	70.3 (7.9)	-	-	-	-
	Unknown	201	100	29	8.7 (2.0)	0	0	-	-	-	-
Prescription medication	Yes	38	15.7 (3.4)	38	19.9 (3.2)	59	75.5 (4.6)	-	-	-	-
	No	163	84.3 (3.4)	196	80.1 (3.2)	37	24.5 (4.6)	-	-	-	-
MEC visit timing	Winter	95	43.0 (6.3)	115	45.3 (6.8)	43	38.4 (7.5)	-	-	-	-
	Summer	106	57.0 (6.3)	119	54.7 (6.8)	53	61.6 (7.5)	-	-	-	-
Female		132		305		98		-		-	
Body weight, kg	<70	116	83.2 (4.3)	166	44.9 (4.1)	60	49.5 (6.5)	-	-	-	-
	70–79	15	16.4 (4.3)	127	49.4 (4.5)	37	48.9 (6.7)	-	-	-	-
	80–94	1	0.4 (0.4)	12	5.7 (2.0)	1	1.6 (1.6)	-	-	-	-
	≥95	0	0	0	0	0	0	-	-	-	-
Race–ethnicity	White	47	65.3 (5.2)	139	69.4 (4.2)	52	82.1 (3.8)	-	-	-	-
	Hispanic	40	15.9 (3.7)	68	10.7 (1.8)	13	3.5 (1.0)	-	-	-	-
	Black	28	11.4 (3.1)	40	7.2 (1.6)	16	6.2 (1.8)	-	-	-	-
	Other	17	7.4 (2.6)	58	12.7 (2.7)	17	8.3 (3.4)	-	-	-	-
Activity METs, min/wk	<1000	48	26.7 (5.0)	120	32.9 (2.5)	51	40.3 (6.5)	-	-	-	-
	1000–4999	65	54.5 (5.4)	130	47.4 (3.9)	39	53.3 (7.3)	-	-	-	-
	≥5000	19	18.8 (4.2)	55	20.0 (2.8)	8	6.3 (2.8)	-	-	-	-
Dietary solute, mOsm/d	<500	55	39.4 (5.0)	99	29.2 (2.9)	44	39.1 (5.6)	-	-	-	-
	500–999	69	56.5 (5.0)	185	65.1 (3.5)	46	54.4 (6.0)	-	-	-	-
	1000–1499	8	4.1 (1.9)	20	5.5 (1.4)	8	6.5 (1.6)	-	-	-	-
	≥1500	0	0	1	0.2 (0.2)	0	0	-	-	-	-
Current cigarette smoker	Yes	-	-	39	14.3 (2.6)	12	13.1 (6.2)	-	-	-	-
	No	-	-	249	83.2 (3.1)	86	86.9 (6.2)	-	-	-	-
	Unknown	132	100	17	2.4 (0.9)	0	0	-	-	-	-
Prescription medication	Yes	38	33.6 (6.2)	130	52.1 (3.3)	68	72.8 (7.1)	-	-	-	-
	No	94	66.4 (6.2)	175	47.9 (3.3)	30	27.2 (7.1)	-	-	-	-
MEC visit timing	Winter	61	31.2 (6.5)	126	36.0 (5.3)	39	37.6 (5.8)	-	-	-	-
	Summer	71	68.8 (6.5)	179	64.0 (5.3)	59	62.4 (5.8)	-	-	-	-

Data representative of the US population were obtained from the 2009–2012 National Health and Nutrition Examination Surveys (NHANES). The sub-group excluded individuals who were older than 70 years, had not fasted for 8 hours, pregnant or lactating women, individuals who self-reported ever having a diagnosis of diabetes or weak or failing kidneys, individuals with a measured BMI outside the normal weight range, and individuals with fasting HgA1c ≥ 6.5%, fasting HOMA-IR ≥ 2.5, fasting glucose ≥ 126 mg/dL, fasting triglyceride ≥ 150 mg/dL, fasting HDL < 40 mg/dL for men, HDL < 50 mg/dL for women, and/or estimated glomerular filtration rate (eGFR) < 60. *n*, Number of individuals who participated in the 2009–2012 NHANES. Weighted %, Represents the weighted proportion of the age group with the row characteristic (Column percent). Activity METs represents physical activity metabolic equivalents. Dietary solute load was calculated from protein, sodium, and potassium intake for the previous day. Prescription medication reflects any prescription medication used in the month before NHANES participation. MEC, Mobile Examination Clinic; Winter timing, Nov–April; Summer timing, May–Oct. Participants that had one or more chronic disease risk factor had a measured BMI outside the normal weight range and/or a measured HgA1c ≥ 6.5%, HOMA-IR ≥ 2.5, fasting glucose ≥ 126 mg/dL, fasting triglyceride ≥150 mg/dL, HDL<40 mg/dL for men, HDL<50 mg/dL for women, and/or CKD-EPI estimated glomerular filtration rate (eGFR) < 60. *Risk factor measurements were only assessed for participants who fasted for 8 hours or longer.
